# The group A *Streptococcus* accessory protein RocA: regulatory activity, interacting partners and influence on disease potential

**DOI:** 10.1111/mmi.14410

**Published:** 2019-11-11

**Authors:** Ira Jain, Jessica L. Danger, Cameron Burgess, Timsy Uppal, Paul Sumby

**Affiliations:** ^1^ Department of Microbiology & Immunology University of Nevada, Reno School of Medicine Reno Nevada USA

## Abstract

The group A *Streptococcus* (GAS) causes diseases that range from mild (e.g. pharyngitis) to severely invasive (e.g. necrotizing fasciitis). Strain‐ and serotype‐specific differences influence the ability of isolates to cause individual diseases. At the center of this variability is the CovR/S two‐component system and the accessory protein RocA. Through incompletely defined mechanisms, CovR/S and RocA repress the expression of more than a dozen immunomodulatory virulence factors. Alleviation of this repression is selected for during invasive infections, leading to the recovery of *covR*, *covS* or *rocA* mutant strains. Here, we investigated how RocA promotes CovR/S activity, identifying that RocA is a pseudokinase that interacts with CovS. Disruption of CovS kinase or phosphatase activities abolishes RocA function, consistent with RocA acting through the modulation of CovS activity. We also identified, in conflict with a previous study, that the RocA regulon includes the secreted protease‐encoding gene *speB*. Finally, we discovered an inverse correlation between the virulence of wild‐type, *rocA* mutant, *covS* mutant and *covR* mutant strains during invasive infection and their fitness in an *ex vivo* upper respiratory tract model. Our data inform on mechanisms that control GAS disease potential and provide an explanation for observed strain‐ and serotype‐specific variability in RocA function.

## Graphical abstract

The group A *Streptococcus* is the causative agent of multiple human diseases. However, the relative ability of isolates to cause individual diseases can differ dramatically. Here, we provide molecular insights into why isolates show such variability and the disease‐specific consequences of it.

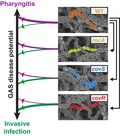

## Introduction

The group A *Streptococcus* (GAS; *Streptococcus pyogenes*) causes a range of human diseases, from self‐limiting pharyngitis (aka strep throat) to the often lethal necrotizing fasciitis (aka the flesh‐eating infection) (Cunningham, 2000). Due to divergent disease manifestations, and significant genome sequence data, GAS has become a model organism to investigate how the virulence and disease potential of a pathogen is influenced by differences in gene content and expression (Sumby *et al.*, 2005; Olsen *et al.*, 2012; Hondorp *et al.*, 2013; Lynskey *et al.*, 2013; Nasser *et al.*, 2014; Miller *et al.*, 2015; Do *et al.*, 2017; Port *et al.*, 2017; Kachroo *et al.*, 2019). Multiple standalone transcriptional regulators (McIver, 2009), two‐component systems (Vega *et al.*, 2016), quorum sensing systems (Jimenez and Federle, 2014) and small regulatory RNAs (Miller *et al.*, 2014) have been characterized, and together they form an interconnecting web of regulatory networks that influence virulence gene expression (Kreikemeyer *et al.*, 2003; Sarkar and Sumby, 2017).

The best‐described two‐component system in GAS is the control of virulence (Cov) system, also known as the capsule synthesis regulatory (Csr) system, which consists of the membrane‐spanning sensor kinase CovS and the response regulator CovR (Levin and Wessels, 1998; Federle *et al.*, 1999). CovR/S negatively regulate the expression of more than 10% of all GAS genes, including many that encode key GAS virulence factors (e.g. the hemolysin streptolysin O, the thrombolytic agent streptokinase, the chemokine protease SpyCEP, the protease SpeB and the anti‐phagocytic hyaluronic acid capsule) (Graham *et al.*, 2002; Sumby *et al.*, 2006; Gryllos *et al.*, 2007). CovS functions as both a kinase and a phosphatase, with these opposing activities altering the ratio of phosphorylated to non‐phosphorylated CovR, which impacts regulation as most CovR/S‐regulated promoters, are repressed by the phosphorylated form of CovR (Gusa *et al.*, 2006; Churchward, 2007; Horstmann *et al.*, 2017).

Modulation of gene expression by CovR/S directly influences GAS disease potential (Graham *et al.*, 2002; Cole *et al.*, 2006; Hollands *et al.*, 2010). In part, the phenotypic consequences of CovR/S‐mediated regulation were identified from studies discovering that *covR* mutant strains and *covS* mutant strains readily arise during invasive infections (Sumby *et al.*, 2006; Yoshida *et al.*, 2015). Such mutant strains are positively selected for due to their upregulation of immunomodulatory proteins (e.g. capsule, SpyCEP) providing increased protection against neutrophil‐mediated killing (Sumby *et al.*, 2008; Li *et al.*, 2014). Interestingly, while most CovR/S‐regulated virulence factors show increased expression in both *covS* or *covR* mutant strains some, such as SpeB, are increased in expression in *covR* mutant strains but are strongly repressed in *covS* mutant strains (Trevino *et al.*, 2009; Feng *et al.*, 2016). Molecular explanations for this additional layer of complexity in the CovR/S system, relative to most described two‐component systems, remain a work‐in‐progress. However, recent data are consistent with the non‐phosphorylated form of CovR being the ‘active’ repressor form with regard to *speB* expression (Chiang‐Ni *et al.*, 2019).

The CovR/S system requires an accessory protein, termed regulator of cov (RocA), for appreciable regulatory activity (Biswas and Scott, 2003). For example, CovR/S represses capsule expression 200‐fold in the presence of RocA but only 4‐fold in its absence (Jain *et al.*, 2017). Similar to the selection of *cov* mutant strains during invasive infections, *rocA* mutant derivatives can also be recovered from parental strains (Feng *et al*., 2016). Interestingly however, in addition to strain‐specific variability in RocA activity, there is also serotype‐specific variability, with some serotypes (M3 and M18) existing exclusively of *rocA* mutant strains (Lynskey *et al.*, 2013; Miller *et al.*, 2015), an observation, that is, not seen with *covS* or *covR* mutant strains.

RocA has homology to membrane‐spanning sensor kinases, although it is unclear whether RocA has kinase activity or rather is a pseudokinase (Lynskey *et al.*, 2013; Jain *et al*., 2017; Sarkar *et al.*, 2018). The regulatory activity of RocA occurs through its ability to enhance CovR/S system function (Miller *et al.*, 2015). While the underlying mechanism is unknown, research identifying that RocA enhances the ratio of phosphorylated to unphosphorylated CovR (Miller *et al.*, 2015) has led to the hypothesis that RocA interacts with CovS, and that this enhances CovS kinase activity toward CovR (Feng *et al*., 2016; Jain *et al.*, 2017).

Here, we present data showing that RocA and CovS interact in the GAS cell membrane, and that both the kinase and phosphatase activities of CovS are required for RocA to show regulatory activity. We also confirm that RocA is a pseudokinase and, contrary to a published report (Feng *et al*., 2016), that RocA is a significant regulator of SpeB expression (which, in part, we show by performing the first analysis of the RocA regulon during stationary phase GAS growth). Finally, while *rocA* mutant, *covS* mutant and *covR* mutant GAS strains can all arise spontaneously during invasive GAS infections we show, through use of competition assays, that these mutations differentially alter the ability of GAS to survive and proliferate in an *ex vivo* model of upper respiratory tract infection. This final finding provides a phenotypic explanation for why we observe serotype‐specific variability in RocA function.

## Results

### An alanine substitution of the predicted RocA auto‐phosphorylation histidine, H246, does not impact RocA activity

**Figure 1:**
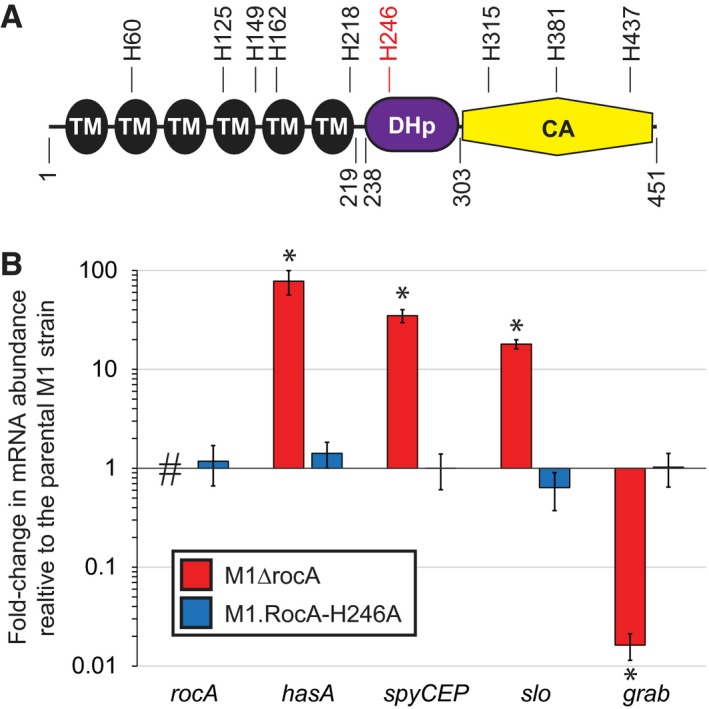
RocA is a pseudokinase, as evident by its predicted auto‐phosphorylation histidine, H246, not being required for activity. A. Domain analysis of RocA. The sensory domain spans from amino acids 1–219 and contains six putative transmembrane (TM) domains. The C‐terminal domain can be divided into two subdomains: the dimerization and histidine phosphotransfer (DHp) domain spans from 238–303; the catalytic (CA) domain spans from 303 to the C‐terminal end. The locations of all nine histidine residues within RocA are highlighted, including the predicted auto‐phosphorylation histidine, H246, located within the DHp domain. B. Taqman‐based quantitative RT‐PCR data showing that RocA function is unaffected by an H246A substitution. The parental serotype M1 GAS strain MGAS2221, *rocA* deletion mutant derivative M1ΔrocA and *rocA* H246A mutant derivative M1.RocA‐H246A were compared. The abundance of the indicated RocA‐regulated mRNAs were determined from triplicate exponential phase GAS cultures that were ran in duplicate, with mean (±standard deviation) shown. The hashtag highlights the lack of *rocA* transcript in the *rocA* deletion mutant strain. The asterisks (*) highlight statistical significance relative to the parental M1 isolate (*T*‐test, *P* < 0.01). [Colour figure can be viewed at https://wileyonlinelibrary.com]

It has been hypothesized that RocA, while having homology to sensor kinases, lacks kinase activity and hence is a pseudokinase (Lynskey *et al.*, 2013; Jain *et al.*, 2017). Sensor kinases function by phosphorylating response regulator proteins to modulate their activity (Capra and Laub, 2012). This phosphorylation event begins with the sensor kinase, usually in the form of a homodimer, auto‐phosphorylating itself on a conserved histidine residue within each monomer, with this phosphate then being transferred to a specific response regulator (Casino *et al.*, 2014). Thus, the conserved histidine residue is critical to sensor kinase activity, and indeed, substituting this histidine is a commonly used method to create inactive derivatives (Goodman *et al.*, 2009; Devi *et al.*, 2015). Based upon amino acid sequence conservation, if RocA auto‐phosphorylates itself then this is predicted to occur at amino acid H246 (Fig. 1A). This prediction is because, although there are nine histidine residues within RocA, H246 is the only one located within the putative dimerization and histidine phosphotransfer domain (DHp), in which the auto‐phosphorylation histidine resides for those sensor kinases that have been described (Stewart, 2010). We set out to test the importance of H246 to RocA function. If a RocA derivative harboring a H246 substitution loses regulatory activity this would be consistent with RocA being a kinase, whereas if a H246 substitution has no effect on regulatory activity this would be consistent with RocA being a pseudokinase. We tested this by creating strain M1.RocA‐H246A, which harbors an H246A substitution in RocA and was created via allelic exchange. Taqman‐based quantitative RT‐PCR was used to compare the expression of several RocA‐regulated genes in a parental M1 strain, a *rocA* deletion mutant derivative (M1ΔrocA) and strain M1.RocA‐H246A. The H246A RocA mutant strain was indistinguishable from the parental isolate (Fig. 1B). Thus, H246 is dispensable for RocA regulatory activity, consistent with RocA being a pseudokinase.

### The kinase and phosphatase activities of CovS are required for RocA‐mediated regulation

**Figure 2:**
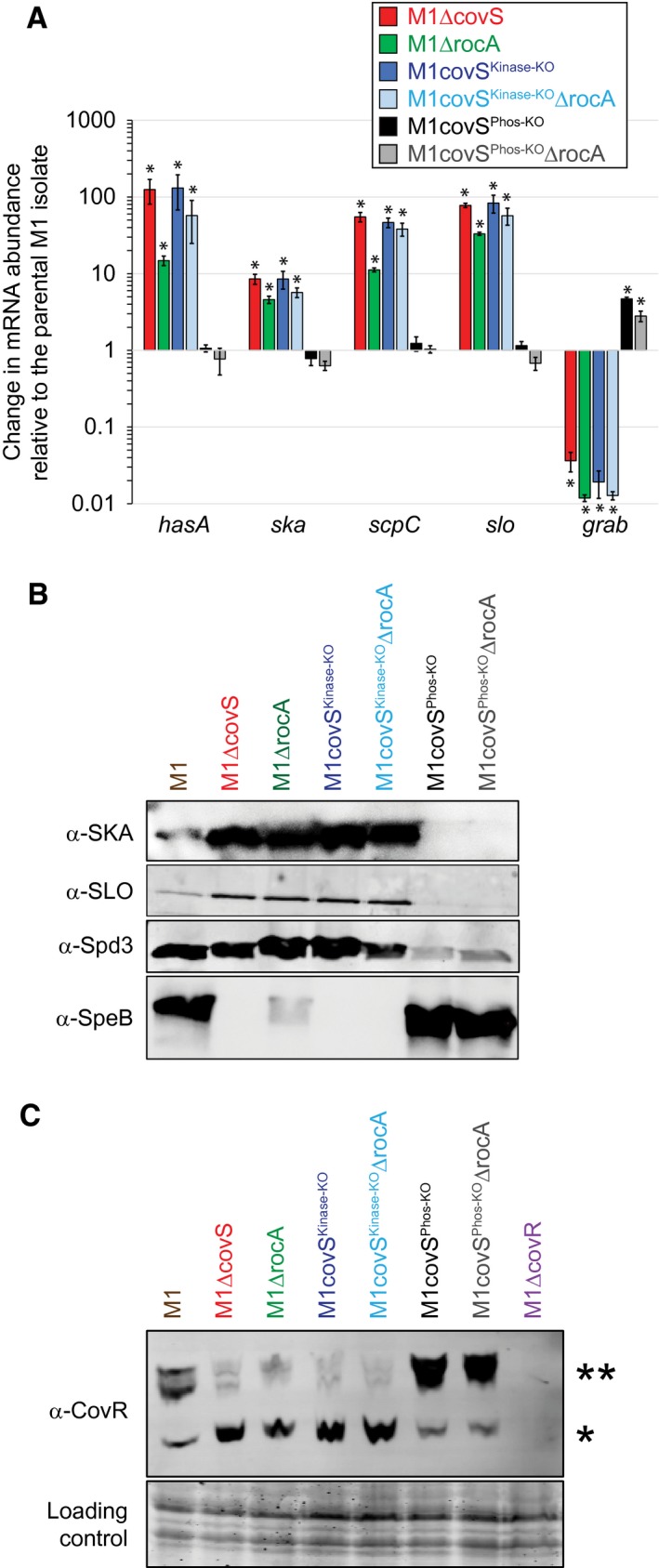
RocA has no regulatory activity in the absence of either the CovS kinase or phosphatase activity. A. Taqman‐based quantitative RT‐PCR analysis. Parental (M1), *covS* deletion mutant (M1ΔcovS), *rocA* deletion mutant (M1ΔrocA), *covS* kinase mutant (M1covS^Kinase‐KO^), *covS* phosphatase mutant (M1covS^Phos‐KO^) and *rocA* deletion mutant derivatives of the CovS kinase (M1covS^Kinase‐KO^ΔrocA) and phosphatase (M1covS^Phos‐KO^ΔrocA) mutant strains were compared. Shown are the averages (±standard deviation) of triplicate samples ran in duplicate. The asterisks highlight statistical significance relative to the parental isolate (*T*‐test, *P < 0.01). B. Western blot analyses showing the absence of any consequences to *rocA* mutation in either M1covS^Kinase‐KO^ or M1covS^Phos‐KO^ strain backgrounds. Antibodies against streptokinase (SKA), streptolysin O (SLO), *S. pyogenes* DNase 3 (Spd3) and the secreted GAS protease (SpeB) were used in conjugation with secreted protein samples recovered from exponential (SKA, SLO, Spd3) or stationary (SpeB) phase cultures of the indicated strains. C. Phos‐Tag analysis showing that RocA does not influence the phosphorylation status of CovR in the absence of CovS kinase or phosphatase activities. Cytoplasmic protein fractions were isolated from the indicated strains and subjected to Phos‐Tag gel electrophoresis followed by Western blot. The bands corresponding to non‐phosphorylated (*) and phosphorylated (**) CovR protein are highlighted. The protein gel was stained and photographed prior to Western analysis to serve as a loading control. [Colour figure can be viewed at https://wileyonlinelibrary.com]

Having previously established that CovS is required for a RocA‐mediated increase in the abundance of phosphorylated CovR (Miller *et al.*, 2015; Jain *et al*., 2017), we sought to investigate whether individual CovS activities (kinase or phosphatase) are needed to observe RocA‐mediated regulation. To this end, we constructed isoallelic derivative strains of a parental serotype M1 strain that express CovS proteins specifically lacking in kinase (CovS^E281A^; strain M1covS^Kinase‐KO^) or phosphatase (CovS^T284A^; strain M1covS^Phos‐KO^) activity. That the E281A and T284A mutations abolish the kinase and phosphatase activities of CovS, respectively, was determined previously (Horstmann *et al.*, 2015). To analyze the regulatory activity of RocA in these backgrounds, *rocA* deletion mutant derivatives of M1covS^Kinase‐KO^ and M1covS^Phos‐KO^ were also constructed. GAS strains were compared by quantitative RT‐PCR and Western blot analyses of select CovR/S and RocA‐regulated genes/proteins, and also by Phos‐Tag Western blot analysis to monitor CovR phosphorylation status. The kinase‐deficient *covS* mutant strain (M1covS^Kinase‐KO^) had a regulatory pattern similar to that of a *covS* deletion mutant strain (Fig. 2A and B), and this was consistent with both strains producing limited amounts of phosphorylated CovR (CovR~P; Fig. 2C). The phosphatase‐deficient *covS* mutant strain (M1covS^Phos‐KO^) had a regulatory pattern that was most similar to that of the parental strain (Fig. 2A and B), consistent with both strains producing high levels of CovR~P (Fig. 2C). Note that while strain M1covS^Phos‐KO^ produces higher levels of CovR~P than the parental strain the *hasA*, *ska*, *spyCEP* and *slo* genes are already maximally repressed in the parental strain (Jain *et al.*, 2017), and hence the additional CovR~P in strain M1covS^Phos‐KO^ has no regulatory consequence for these genes (Fig. 2A). There are, however, consequences at the protein level for the increased levels of CovR~P in strain M1covS^Phos‐KO^ (Fig. 2B), which we propose are due to the elevated level of the secreted protease SpeB in M1covS^Phos‐KO^ resulting in increased proteolysis of SKA, SLO and Spd3. While transcription from the *hasA*, *ska*, *spyCEP* and *slo* genes is not affected by the higher level of CovR~P in strain M1covS^Phos‐KO^, there is a ~5‐fold increase in the abundance of *grab* mRNA (Fig. 2A). This is consistent with our hypothesis that *grab*, similar to *speB*, is negatively regulated by non‐phosphorylated rather than phosphorylated CovR (Jain *et al*., 2017). The key finding from the data in Fig. 2 is that RocA has no regulatory activity in the absence of either CovS kinase or phosphatase activities (compare M1covS^Kinase‐KO^ and M1covS^Phos‐KO^ with their *rocA* deletion mutant derivatives).

### Stk is dispensable for RocA activity

**Figure 3:**
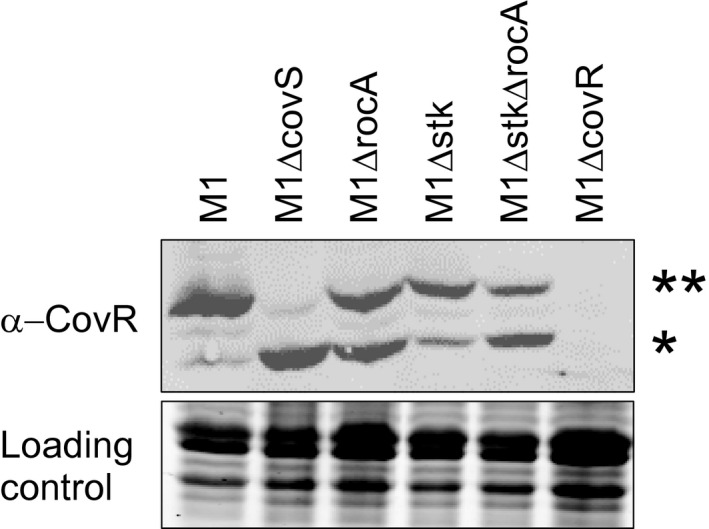
RocA retains regulatory activity in the absence of the eukaryotic‐like serine/threonine kinase Stk. Phos‐tag Western blot analysis. The bands corresponding to non‐phosphorylated (*) and phosphorylated (**) CovR protein are highlighted. The protein gel was stained and photographed prior to Western analysis to serve as a loading control.

CovS, which phosphorylates CovR at amino acid D53, is not the only GAS kinase that has been reported to phosphorylate CovR. The serine‐threonine kinase (Stk) phosphorylates CovR at amino acid T65, and this precludes phosphorylation of D53, and hence activation, by CovS (Horstmann *et al.*, 2014). It is therefore possible that RocA modulates CovS activity indirectly, by reducing Stk kinase activity toward CovR. To test this, we created *stk* mutant (M1Δ*stk*) and *stk/rocA* double mutant (M1Δ*stk*Δ*rocA)* derivatives of our parental M1 GAS isolate and assessed whether RocA retained regulatory activity in the absence of Stk. Using Phos‐Tag Western blot analysis of CovR phosphorylation status we observed that RocA enhanced the ratio of CovR~P to CovR regardless of the presence or absence of Stk (Fig. 3). Thus, modification of CovS activity by RocA does not occur indirectly through the inhibition of Stk.

### Immunofluorescence data are consistent with the co‐localization of RocA and CovS

**Figure 4:**
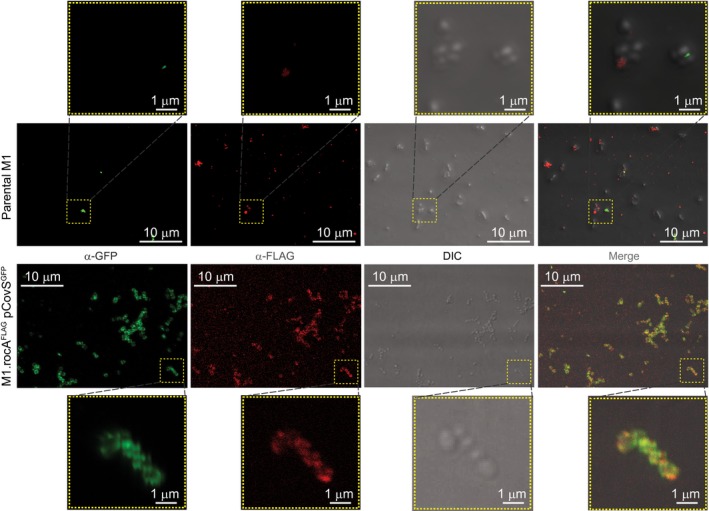
Immunofluorescence microscopy data are consistent with the co‐localization of RocA and CovS. Bacteria from exponential phase cultures of parental and RocA/CovS‐tagged (M1.rocA^FLAG^ pCovS^GFP^) GAS strains were fixed on glass coverslips, permeabilized and stained with GFP‐ and FLAG‐specific antibodies. Images were obtained using a laser scanning confocal microscope (Carl Zeiss, Inc.) and processed using ZEN imaging software (Carl Zeiss, Inc.) to assign the appropriate color. Different panels were magnified (yellow boxes) to demonstrate co‐localization of the tagged‐proteins. Co‐localization of RocA (FLAG‐tagged; red) and CovS (GFP‐tagged; green) is highlighted by yellow signals in the merge panel of strain M1.rocA^FLAG^ pCovS^GFP^. A scale, in µm, is shown for each image.

As previously discussed (Jain *et al.*, 2017), we propose that RocA and CovS interact through their respective membrane‐spanning regions, and that this interaction enhances CovS kinase activity. In the first of a three‐pronged approach that we undertook to investigate putative interactions between RocA and CovS we utilized immunofluorescence microscopy. We generated a strain, M1.rocA^FLAG^ pCovS^GFP^, which expresses derivatives of RocA and CovS that are FLAG‐ and GFP‐tagged, respectively. Cells from exponential phase cultures of M1.rocA^FLAG^ pCovS^GFP^, along with the parental strain MGAS2221 (as a negative control), were permeabilized and anti‐FLAG and anti‐GFP antibodies were added. All cells of strain M1.rocA^FLAG^ pCovS^GFP^ gave strong levels of signal with both antibodies, whereas signal from parental strain cells was sporadic (Fig. 4). While the resolution of the images is insufficient to show direct molecular interactions between RocA and CovS, overlapping signal foci for strain M1.rocA^FLAG^ pCovS^GFP^, represented by yellow signals in the merged image, are consistent with this hypothesis (Fig. 4).

### A bacterial two‐hybrid approach identifies interaction between CovS and CovR, but does not identify interaction between CovS and RocA

In the second of our three‐pronged approach to investigate putative interactions between CovS and RocA we used a bacterial two‐hybrid (BACTH) system. We used an *E. coli*‐based BACTH system in which test proteins are fused with fragments T18 or T25 of adenylate cyclase (CyaA) (Karimova *et al.*, 1998; Battesti and Bouveret, 2012), such that if the test proteins interact, they bring the T18 and T25 fragments together creating a functional protein, which is detected via standard β‐galactosidase assays. Importantly, this system has been successfully used to detect interactions between the *S. agalactiae* CovR/S proteins, and between *S. agalactiae* CovS and the membrane‐spanning Abi‐domain protein Abx1 (Firon *et al.*, 2013). Note that Abx1 is not encoded by GAS, and in contrast to RocA, is a negative regulator of CovR/S function (Firon *et al.*, 2013). Plasmids expressing N‐terminal T25‐tagged RocA, CovS or CovR proteins, and similar plasmids expressing N‐terminal T18‐tagged RocA, CovS or CovR proteins, were constructed.

**Figure 5:**
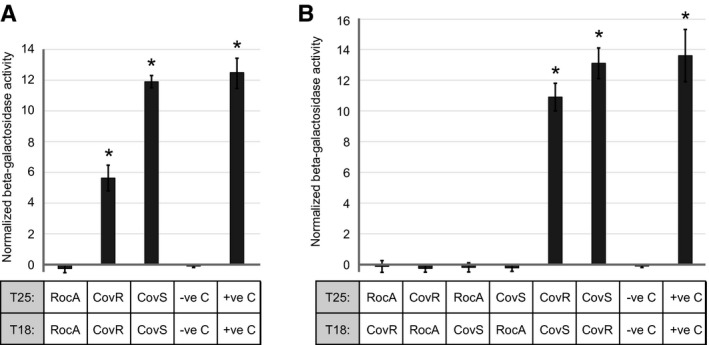
A bacterial two‐hybrid approach identifies protein: protein interactions between CovS and CovR, but not between RocA and either Cov protein. RocA, CovR and CovS fusion proteins were constructed with the T18 or T25 fragments of adenylate cyclase. T18‐ and T25‐based plasmids were co‐transformed into *E. coli* and the activity of the β‐galactosidase cAMP‐dependent reporter gene was determined. Shown are the means (± standard deviation) calculated from two independent cultures ran in triplicate. The asterisks (*) highlight statistical significance relative to the negative control strain (‐ve C; which contains empty T18 and T25 vectors) (*T*‐test, *P* < 0.01). A positive control strain (+ve C) was used in which the T18 and T25 fragments were made to interact through the addition of leucine zippers. A. Assays of homo‐dimerization. B. Assays of interactions between RocA, CovR and/or CovS.

We began by investigating whether CovR, CovS and/or RocA can form homodimers. The two RocA‐, CovS‐ or CovR‐based plasmids were co‐transformed into the *E. coli* strain BTH101, and the resultant strains were compared for β‐galactosidase activity. Our results confirm previous data that the GAS CovR protein is able to form homodimers (Gusa *et al.*, 2006), as evident by the strong β‐galactosidase activity of the *E. coli* strain containing pT25‐CovR and pT18‐CovR (Fig. 5A), and also provide the first experimental data that the GAS CovS protein forms homodimers (Fig. 5A). The *E. coli* strain containing both RocA fusion proteins did not show any appreciable β‐galactosidase activity (Fig. 5A).

Next, by co‐transforming different combinations of plasmids, we tested whether protein: protein interactions occur between CovR, CovS and/or RocA. That CovR and CovS interact was readily apparent from our β‐galactosidase data (Fig. 5B), and while long expected, this is the first experimental data in GAS that confirms this. However, no interaction was observed between RocA and either Cov protein (Fig. 5B), and hence our hypothesis, that RocA interacts with CovS, is not supported by the data. Unfortunately, since the BACTH method is performed in *E. coli* it is not possible to assess whether the RocA fusion proteins (with T18/T25) are active. Thus, it may be that the lack of interaction between RocA and CovS, as well as the lack of identification of RocA homodimers, is a consequence of limitations of the BACTH system rather than the true absence of any interaction.

### RocA co‐immunoprecipitates with CovS

**Figure 6:**
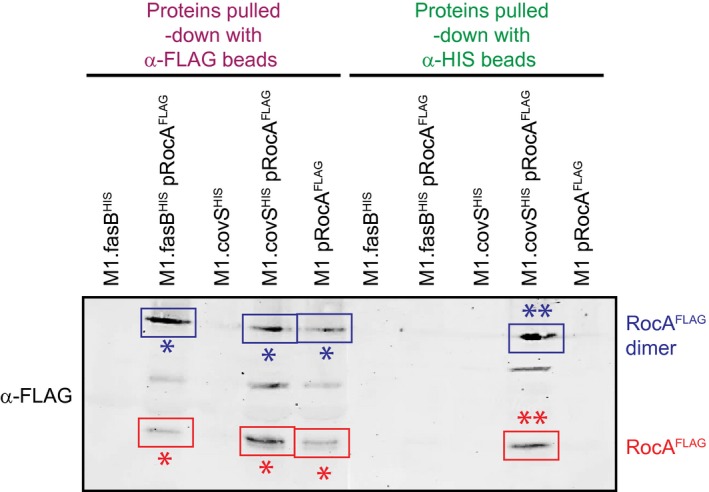
Co‐immunoprecipitation of RocA with CovS. Co‐IP data generated using membrane protein fractions recovered from GAS strains that express chromosomally‐ and/or plasmid‐encoded FLAG‐tagged RocA, HIS‐tagged CovS and/or HIS‐tagged FasB (used as a negative control to guard against the non‐specific pull‐down of proteins). Membrane proteins were used in pull‐down assays in conjugation with anti‐FLAG (left five lanes) or anti‐HIS (right five lanes) beads, with the recovered proteins being used in Western analysis with an anti‐FLAG antibody. Detected RocA^FLAG^ proteins, both monomers and dimers, are highlighted with boxes. Asterisks signify immunoprecipitated (*) and co‐immunoprecipitated (**) proteins. [Colour figure can be viewed at https://wileyonlinelibrary.com]

The third of our three‐pronged approach to investigate possible interactions between CovS and RocA made use of co‐immunoprecipitation (co‐IP). We hypothesized that antibodies against CovS would pulldown not only CovS from GAS membrane protein fractions but also, due to protein: protein interactions, RocA. To facilitate testing this hypothesis we created derivatives of our parental M1 GAS isolate that harbored single‐ (M1.fasB^HIS^, M1.covS^HIS^, M1 pRocA^FLAG^) or double‐ (M1.fasB^HIS^ pRocA^FLAG^, M1.covS^HIS^ pRocA^FLAG^) tagged proteins. Note that FasB is a membrane‐spanning sensor kinase, that is, part of a three‐component system that is not known to interact with CovS, CovR or RocA (Ramirez‐Pena *et al.*, 2010). The FasB‐tagged strain was created for use as a negative control to guard against non‐specific co‐IP. GAS membrane protein fractions from each of the single‐ and double‐tagged strains were isolated and split, with half of each sample being used in conjunction with anti‐FLAG beads (to pulldown RocA and any potential binding partners) and the other half with anti‐HIS beads (to pulldown CovS and any potential binding partners). Recovered proteins were subsequently subjected to Western blot analysis, probing with anti‐FLAG antibodies. As hypothesized, the pulldown of CovS using anti‐HIS beads with proteins from strain M1.covS^HIS^ pRocA^FLAG^ resulted in the co‐IP of RocA (Fig. 6). The data are consistent with CovS and RocA interacting in the GAS cell membrane. See the Discussion section for a summary of our thoughts with regard to CovS: RocA interactions.

### RocA is a positive regulator of SpeB expression

Due to the wide diversity of substrates cleaved, which include both host (e.g. IgG and C3b) and GAS (e.g. streptolysin O and streptokinase) proteins, the secreted protease SpeB is a major GAS virulence factor (Nelson *et al.*, 2011). SpeB is primarily expressed during stationary phase growth, with the RopB‐based quorum sensing system (Neely *et al.*, 2003; Do *et al.*, 2017), and the CovR/S two‐component system (Federle *et al.*, 1999; Trevino *et al.*, 2009), playing key regulatory roles. The regulation of SpeB expression by CovR/S is atypical for a two‐component system in that *covR* mutant and *covS* mutant strains have different regulatory consequences. A *covR* mutant strain increases SpeB expression ~3‐fold relative to the parental strain, while a *covS* mutant strain decreases expression ~500‐fold (Trevino *et al.*, 2009). This and other data has led to the generation of a model of CovR/S‐mediated expression in which the majority of regulated genes are repressed by CovR~P, but a subset (e.g. *speB* and *grab*) are repressed by unphosphorylated CovR (Trevino *et al.*, 2009; Sarkar and Sumby, 2017). A recent publication supports the negative regulation of *speB* expression by unphosphorylated CovR (Chiang‐Ni *et al.*, 2019).

**Figure 7:**
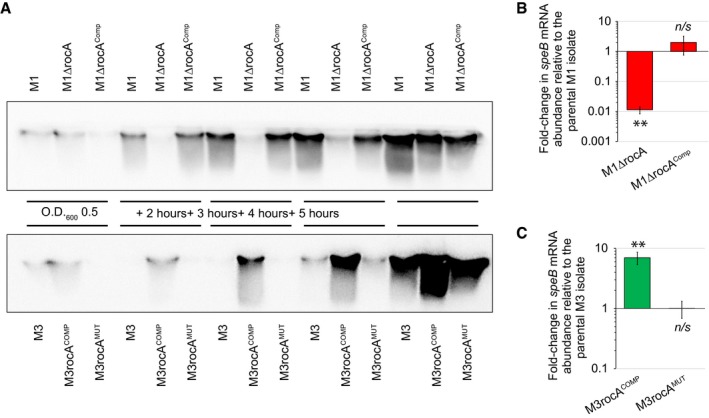
RocA alters the growth phase‐regulated expression of the secreted protease SpeB. A. Western blot analysis of SpeB expression by the indicated strains recovered from mid‐exponential phase (O.D.600 = 0.5) and early‐to‐late stationary phase (2, 3, 4 and 5 h after reaching O.D.600 = 0.5) cultures. B and C. Quantitative RT‐PCR was performed using RNA isolated from triplicate mid‐stationary phase (3 h after reaching an O.D.600 of 0.5) cultures of each strain. Panel B shows the data gained from M1 GAS isolates. Panel C shows the data gained from M3 GAS isolates. Shown is the average (± standard deviation) from two independent experiments. Statistical significance was tested via the Wilcoxon Signed Rank Test, **P < 0.0001, n/s = not significant.

Working in a serotype M1 GAS strain background, it was reported that RocA has no regulatory activity toward SpeB expression (Feng *et al*., 2016). However, given that CovR/S have a central role in regulating SpeB expression, and that RocA has a central role in modifying CovR/S activity, we hypothesized that RocA does in fact regulate SpeB levels. In support of this, a recent RNAseq‐based transcriptome analysis, performed in a serotype M28 strain background, identified *speB* as being positively regulated by RocA (Bernard *et al.*, 2018). Our SpeB Western data from Fig. 2B, which shows reduced expression in the *rocA* mutant strain, was the first piece of data that we generated in support of our hypothesis that RocA regulates SpeB expression, even in serotype M1 strain backgrounds. To comprehensively assay the role of RocA in SpeB expression, we isolated secreted protein samples from GAS cultures grown from the exponential phase through to late stationary phase. GAS strains used in this study were a mixture of serotype M1 and M3 strains. The M1 strains consisted of a parental isolate (M1), a *rocA* mutant derivative (M1ΔrocA), and a complemented mutant derivative (M1ΔrocA^Comp^). The M3 strains consisted of a parental isolate (M3; which, like all M3 isolates (Miller *et al.*, 2015), is naturally *rocA* mutant), a derivative of the parental isolate in which the *rocA* mutation has been complemented (M3rocA^COMP^), and a derivative of strain M3rocA^COMP^ in which the *rocA* gene has been re‐mutated so that it mirrors that seen in the parental strain (M3rocA^MUT^). Regardless of the serotype, isolates harboring functional *rocA* alleles produced higher levels of SpeB than *rocA* mutant derivatives (Fig. 7A). The difference in SpeB expression is particularly pronounced in mid‐stationary phase samples (3 h after reaching an O.D.600 of 0.5). In contrast, by late stationary phase (5 h after reaching an O.D.600 of 0.5), there is only a negligible difference in SpeB expression levels between the tested strains of each serotype.

To assess whether the regulation by RocA occurs at the RNA level we performed quantitative RT‐PCR analysis using RNA isolated from mid‐stationary phase GAS cultures. For both the M1 (Fig. 7B) and M3 (Fig. 7C) strains we observed that RocA enhances *speB* mRNA abundance. Thus, RocA enhances SpeB expression by increasing the abundance of *speB* mRNA.

### RocA is a major regulator of gene expression in stationary phase GAS

**Figure 8:**
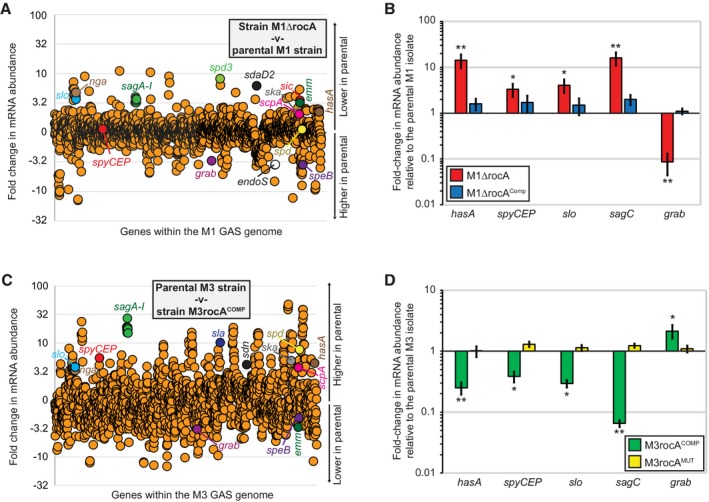
RocA is a major regulator of GAS mRNA transcripts during stationary phase growth. A. Summary of an RNA‐Seq‐based transcriptome comparison between stationary phase cultures of our parental M1 strain (MGAS2221) and *rocA* mutant derivative (M1ΔrocA). The relative expression levels of all genes are graphed, with each represented by a circle. Select virulence factor‐encoding genes are colored and labeled. Genes are arranged in the same order as they appear in the chromosome. B. Taqman‐based verification of select transcripts identified as being differentially regulated in the RNA‐Seq analysis of panel A. The abundance of the indicated mRNAs were determined from triplicate exponential phase GAS cultures of each strain. The experiment was performed in triplicate with mean (± standard deviation) shown. The asterisks highlight statistical significance relative to the parental M1 isolate (T‐test, **P < 0.01, *P < 0.05). C and D. These panels are similar to A and B but were generated by comparing a parental M3 strain (MGAS10870, which is naturally *rocA* mutant) with its *rocA*‐complemented derivative (M3rocA^COMP^).

The RocA regulon during stationary phase growth has not previously been investigated and, given what is known about RocA function, we postulated that *speB* was not the sole gene regulated by this protein. To investigate this on a transcriptome‐wide scale we performed RNA‐Seq‐based transcriptome analyses comparing parental and *rocA* mutant M1 isolates, and also parental and *rocA* complemented M3 isolates. Using a 2‐fold cutoff level, we identified 206 (the M1 data, Fig. 8A) and 713 (the M3 data, Fig. 8C) genes as differing in a statistically significant manner (Kal's Z‐test with a false discovery rate correction) between the presence and absence of RocA during stationary phase growth. While more genes were identified as being RocA‐regulated in the M3 comparison than in the M1 comparison, there is significant overlap in the data (i.e. almost all of the 206 M1 genes are included within the 713 M3 genes), including most of the virulence factor‐encoding genes shown in Fig. 8A and 8. Regulatory differences identified from the RNA‐Seq data were verified, for select virulence factor encoding genes, by quantitative RT‐PCR analyses (Fig. 8B and C). The data are consistent with RocA being a major regulator of GAS gene transcription during the stationary phase of growth.

### Competition assays between parental, *rocA* mutant, *covS* mutant and *covR* mutant GAS strains during growth in human saliva identifies variability

**Figure 9:**
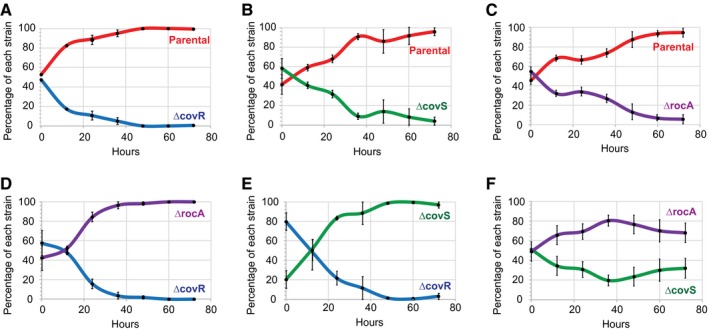
The regulatory disparity between parental, *rocA* mutant, *covS* mutant and *covR* mutant GAS strains impact their ability in an *ex vivo* model of upper respiratory tract infection. Growth competition assays in human saliva were performed between a parental M1 isolate (MGAS2221) and isogenic *covR*, *covS* or *rocA* mutant derivatives. Each pairwise strain comparison was performed a minimum of three times, with the mean and standard deviation shown. All comparisons showed statistically significant differences in growth (*P* < 0.001; repeated measures ANOVA).

The selection of *covR*, *covS* and *rocA* mutant strains during invasive GAS infections has been well established (Engleberg *et al.*, 2001; Sumby *et al.*, 2006; Hollands *et al.*, 2010; Feng *et al*., 2016). It has also been shown that these mutant strains are not identical with regard to their virulence in animal models of invasive infection (Sumby *et al.*, 2006; Ikebe *et al.*, 2010; Li *et al.*, 2014; Yoshida *et al.*, 2015; Feng *et al*., 2016), with the following order of virulence being observed: *covR* mutants > *covS* mutants > *rocA* mutants > parental isolates. It has been proposed that while *covR*, *covS* and *rocA* mutant strains show increased virulence during invasive infections, that this comes at a fitness cost during pharyngeal infections (Trevino *et al.*, 2009; Alam *et al.*, 2013; Sarkar and Sumby, 2017). Indeed, we propose an inverse correlation between invasive disease virulence and fitness during pharyngeal infections (i.e. we hypothesize that for pharyngeal infections the order of virulence is: *covR* mutants < *covS* mutants < *rocA* mutants < parental isolates). As a means to test the relative abilities of such isolates to cause pharyngeal infections we performed competition assays during growth in human saliva, which is a well‐established *ex vivo* model of an upper respiratory tract infection (Shelburne *et al.*, 2005, Trevino *et al.*, 2009, Zhu *et al.*, 2017). All pairwise permutations of our four tested strains were compared for their ability to survive and proliferate in saliva. As hypothesized, the parental isolate outcompeted all three mutant strains (Fig. 9A–C), the *rocA* mutant strain outcompeted the *covR* and *covS* mutant strains (Fig. 9D and 9), and the *covS* mutant strain outcompeted the *covR* mutant strain (Fig. 9E). Thus, our saliva competition data supports the key finding of an inverse correlation between GAS invasive disease virulence and fitness during pharyngeal infections.

## Discussion

The many diseases caused by GAS differ dramatically in their clinical presentation and lethality. Additional layers of variability are observed due to strain‐ and serotype‐specific differences in the ability of isolates to cause distinct diseases, with molecular mechanisms such as the modulation of regulatory systems, or gene gain/loss, accounting for such variation (Cao *et al.*, 2014; Sarkar and Sumby, 2017; Jain *et al.*, 2019; Kachroo *et al.*, 2019). At the center of strain‐ and serotype‐specific variability in GAS disease potential is the CovR/S two‐component system. Disruption of CovR/S activity, either directly through *covR* or *covS* mutation, or indirectly through mutation of the critical accessory protein‐encoding gene *rocA*, is a major driver of strain‐specific variability (Engleberg *et al.*, 2001; Cole *et al.*, 2006; Trevino *et al.*, 2009; Feng *et al*., 2016). Furthermore, due to some serotypes existing exclusively of *rocA* mutant strains (e.g. serotypes M3 and M18), this impacts regulation at a serotype‐specific level (Lynskey *et al.*, 2013; Miller *et al.*, 2015). Here, we investigated RocA activity and identified that (i) RocA is a pseudokinase; (ii) RocA does not influence CovR/S activity indirectly by modulating Stk activity; (iii) disruption of either CovS kinase or phosphatase activity precludes RocA activity; (iv) RocA interacts with CovS within the GAS cell membrane; (v) RocA is a major regulator of gene transcription during stationary phase growth; and (vi) loss of RocA activity results in a reduced ability to compete with wild‐type GAS in an *ex vivo* model of pharyngitis, while retaining the ability to outcompete *covS* or *covR* mutant strains. Our data provide insights into the functioning of a key virulence regulatory system and inform on molecular mechanisms that control GAS disease potential.

Despite the similarity between RocA and membrane‐spanning sensor kinases it was proposed that RocA is a pseudokinase. Evidence for RocA being a pseudokinase included that the putative CA domain of RocA (Fig. 1A) lacks several amino acids that normally contribute to kinase function (Biswas and Scott, 2003), and that overexpression of a truncated form of RocA lacking the CA domain is capable of complementing a *rocA* mutant strain (Jain *et al*., 2017). However, given that overexpression of the truncated RocA protein was required for complementation, it remained a possibility that RocA had kinase activity. We addressed this issue by showing that substitution of the putative auto‐phosphorylation histidine residue within RocA did not impact activity (Fig. 1B), consistent with RocA not requiring auto‐phosphorylation for regulatory activity, and hence that RocA is a pseudokinase.

The lack of kinase activity by RocA indicates that this protein indirectly increases the ratio of phosphorylated to non‐phosphorylated CovR (Miller *et al.*, 2015). One possible mechanism of regulation was that RocA inhibits the activity of Stk, given that this protein negatively regulates the ability of CovR to be phosphorylated by CovS (Horstmann *et al.*, 2014; Kant *et al.*, 2015). We tested this hypothesis after creating single and double *rocA/stk* mutant GAS strains, identifying that RocA retains activity in the absence of Stk (Fig. 3), and hence that RocA does not function through Stk.

Current data are consistent with RocA interacting with CovS in the GAS cell membrane, an interaction that enhances CovS kinase activity toward CovR. Such data include that the antimicrobial peptide LL‐37, which normally inhibits CovS activity (Velarde *et al.*, 2014), is unable to do so in the absence of RocA (Jain *et al*., 2017), and that loss of either the kinase or phosphatase activities of CovS prevent RocA from regulating CovR/S activity (Fig. 2). Furthermore, our immunofluorescence microscopy (Fig. 4) and co‐IP (Fig. 6) data support interactions between RocA and CovS. Importantly, while this work was under initial review a manuscript by Lynskey *et al*. was published in which, using a co‐IP‐based approach, CovS and RocA were shown to interact via their membrane‐spanning domains (Lynskey *et al.*, 2019). They also identified, through a BACTH approach, that RocA can form homodimers. This was particularly interesting to us as while, in our own BACTH approach, we observed homodimerization by CovR and by CovS (Fig. 5A), as well as interactions between CovR and CovS (Fig. 5B), we did not observed RocA homodimerization. We believe that the disparity in the two BACTH datasets with regard to RocA is most likely a consequence of the fact that we used the full‐length RocA protein in our study, while only the C‐terminal (cytoplasmic) half of RocA was used in the Lynskey *et al*. study. It is our contention that the lack of any interactions with RocA (homodimer formation or interactions with CovS) was a consequence of our RocA fusion proteins being non‐functional in our BACTH assay. The hypothesized lack of function for the RocA fusion proteins may be a consequence of their inappropriate expression, folding and/or processing. As RocA has six putative transmembrane (TM) domains, this increases the probability that this protein would not be arranged appropriately in the cell membrane.

Given that RocA plays a key role in promoting the regulatory activity of CovR/S, we were not surprised by the large size of the stationary phase RocA regulon (Fig. 8). However, as this had not been looked at previously, and given the publication of Feng *et al*. that stated that SpeB expression was unaffected by RocA (Feng *et al*., 2016), at least in serotype M1 strains, our transcriptomic analysis fills an important gap in the field. Ultimately, our data shows that RocA influences the growth phase expression of SpeB, such that there is a dramatic reduction in the accumulation of SpeB in a *rocA* mutant strain in the early‐ and mid‐stationary phases of growth (Fig. 7A). However, by late stationary phase (5 h after reaching an O.D.600 of 0.5), SpeB levels are similar regardless of the presence or absence of RocA. This likely accounts for why the study by Feng *et al*. did not identify a regulatory role for RocA against SpeB as they only used a single (late stationary phase) sample in their work (Feng *et al*., 2016). We hypothesize that RocA promotes SpeB expression by increasing CovS kinase activity toward CovR, lowering the concentration of non‐phosphorylated CovR in the cell which, since this is the form of CovR that represses *speB* expression (Chiang‐Ni *et al.*, 2019), results in derepression. To date, it is not known whether the repression of *speB* expression by non‐phosphorylated CovR is direct or indirect. To the best of our knowledge, the regulation of different subsets of genes by phosphorylated and non‐phosphorylated forms of a response regulator has not been described in any other system.

As can be seen from our RNA‐Seq data (Fig. 8A and B), *speB* is only one of more than a dozen virulence factor‐encoding genes that are regulated by RocA in stationary phase. As all serotype M3 and M18 GAS isolates harbor null mutations in *rocA* (Lynskey *et al.*, 2013; Miller *et al.*, 2015), and *rocA* mutants can arise from wild‐type strains during invasive infections (Feng *et al.*, 2016), the regulatory pattern observed in Fig. 8 is displayed by a significant number of clinical isolates, and likely influences GAS: host interactions.

**Figure 10:**
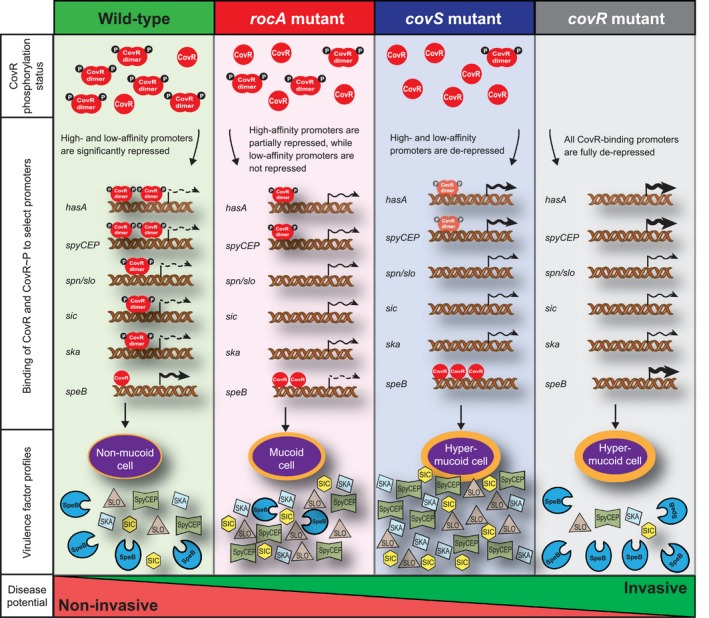
Model showing the varying disease potential and regulatory consequences of mutating individual genes encoding components of the CovR/CovS/RocA regulatory system. The diagram displays what occurs in a GAS cell that harbors a fully functional CovR/CovS/RocA regulatory system (green‐shaded section), in a *rocA* mutant strain (red‐shaded section), in a *covS* mutant strain (blue‐shaded section) and in a *covR* mutant strain (grey‐shaded section). Differences in virulence factor profiles between the strains are a consequence of differences in the ratio of phosphorylated and non‐phosphorylated CovR. Phosphorylated CovR binds to, and represses transcription from, multiple promoter regions within the GAS genome with varying affinity. Level of transcription from individual promoters is highlighted by the format of the transcription line (none, dashed or full) and by the thickness of the transcription line. When phosphorylated CovR is in high abundance (i.e. in a wild‐type strain), there is sufficient for a dramatic reduction in transcription from both high‐ (e.g. *hasA* and *spyCEP*) and low‐(e.g. *spn/slo*, *sic* and *ska*) affinity promoters. Transcription of *speB*, which unlike most CovR‐regulated promoters is regulated by non‐phosphorylated CovR, is modestly repressed by the small amount of non‐phosphorylated CovR available in this strain. When phosphorylated CovR is reduced in abundance (i.e. in a *rocA* null mutant strain), it is sequestered by high affinity promoters, providing moderate negative regulation of these genes but only modest or no regulation of genes that harbor low‐affinity promoters. The greater abundance of non‐phosphorylated CovR in this strain leads to a reduction in *speB* transcription. In the near absence of phosphorylated CovR (i.e. in a *covS* null mutant strain), most CovR‐regulated promoters are fully depressed, with the small amount of phosphorylated CovR available having modest repressive activity at high‐affinity promoters (represented by lighter shading of CovR~P). Note that some phosphorylated CovR is created even in the absence of CovS through the ability of CovR to be phosphorylated at low level by the phosphate donor acetyl phosphate. Transcription of *speB* is fully repressed in this strain background due to the high abundance of non‐phosphorylated CovR. In the absence of CovR (i.e. in a *covR* null mutant strain), all CovR‐regulated promoters are fully depressed, resulting in high gene transcription levels for multiple virulence factor encoding genes. However, due to the proteolytic activity of SpeB, secreted and cell wall anchored virulence factors other than SpeB do not accumulate in high abundance.

The finding that all serotype M3 and M18 GAS isolates harbor null mutations in *rocA* led us to query why no serotypes exist that are exclusively *covS* or *covR* mutant strains? To ask this in another way, what distinguishes a *rocA* mutant strain from a *covS* or *covR* mutant strain? We and others have shown that wild‐type, *rocA* null mutant, *covS* null mutant and *covR* null mutant strains are not equal with regard to invasive disease virulence (Sumby *et al.*, 2006; Ikebe *et al.*, 2010; Li *et al.*, 2014; Yoshida *et al.*, 2015; Feng *et al*., 2016). The order of invasive disease virulence for these strains is wild‐type < *rocA* mutant < *covS* mutant < *covR* mutant. The greater invasive disease virulence of *covR* and *covS* mutant strains also begs the question of why these isolates are not more prevalent in the GAS population? These isolates can be recovered from some invasive infections but are very rarely recovered from pharyngeal infections, which represent the vast majority of GAS infections and are essential to maintaining strains within a population. The competition data shown in Fig. 9 is consistent with the hypothesis that there is an inverse correlation between the ability to cause pharyngeal infections and invasive disease virulence. We propose that the negative affect of *covR* or *covS* mutation on the ability of strains to cause pharyngeal infections is such that they are unable to be maintained in the population. In contrast, the negative impact of *rocA* mutation on the ability to cause pharyngeal infections is not as severe, as evident by the ability of the *rocA* mutant strain to out‐compete the *covS* and *covR* mutant strains (Fig. 9D and E). We propose that this explains why some GAS serotypes can tolerate consisting of exclusively *rocA* mutant strains, as these strains retain a moderate ability to cause pharyngeal infections, but why no GAS serotypes are exclusively *covS* or *covR* mutant strains, as these strains have moved past a tipping point with regard to their ability to efficiently cause pharyngeal infections. Figure 10 shows a model of the regulatory and disease potential differences between wild‐type, *rocA* mutant, *covS* mutant and *covR* mutant strains.

Since *covR* or *covS* mutant strains that spontaneously arise during invasive infections are unlikely to be maintained in the population, why are these strains selected for in the first place? We believe that this is answered by a bystander effect. That is, the mutant strains that arise during infection secrete high levels of immunomodulatory virulence factors that protect not only themselves but also co‐infecting wild‐type GAS cells (Sumby *et al.*, 2006). This enhances the likelihood of the wild‐type GAS disseminating to new hosts.

In summation, we have characterized the key accessory protein RocA, identifying it as a pseudokinase, and verifying that it interacts with the sensor kinase component of the virulence‐regulating two‐component system CovR/S. We also discovered that RocA is a major regulator of virulence gene expression during stationary phase growth, and that the disruption of *covR*, *covS* or *rocA* results in the attenuation of fitness in an *ex vivo* model of upper respiratory tract infection. The greater attenuation of *covS* and *covR* mutant strains, relative to *rocA* mutant strains, provide an explanation for previously observed serotype‐specific variability in RocA activity.

## Experimental procedures

### Strains and culture conditions

The representative serotype M1 and M3 clinical GAS isolates MGAS2221 and MGAS10870, respectively, were used in this study. Information about these strains and their derivatives is present in Table S1. GAS isolates were grown in THY broth, with chloramphenicol (4 μg ml^–1^), kanamycin (200 μg ml^–1^) or spectinomycin (150 μg ml^–1^) added when needed. For standard cloning, DH5α *E. coli* cells were used. For BACTH assays, BTH101 *E. coli* cells were used. *E. coli* were grown in LB broth with agitation at 37°C, with ampicillin (100 μg ml^–1^), kanamycin (50 μg ml^–1^) and chloramphenicol (20 μg ml^–1^) added when needed.

### Creation of strain M1.RocA‐H246A

Allelic replacement was used to substitute the wild‐type *rocA* gene of MGAS2221 for an allele that expresses an H246A RocA derivative. Allele replacement made use of the suicide vector pBBL740 via standard techniques (Ramirez‐Pena *et al.*, 2010; Miller *et al.*, 2015). Briefly, overlapping primers were designed to amplify the *rocA* gene from MGAS2221 while simultaneously making the required nucleotide changes (see Table S2). The PCR products were joined with PCR‐amplified pBBL740 via Gibson Assembly (New England Biolabs), and the resultant plasmids were sequence‐verified. The plasmid was transformed into MGAS2221 competent cells, selecting for chloramphenicol resistance on THY agar plates. The passaging and patching protocol, to switch out the wild‐type *rocA* allele for the mutant alleles, was performed as described (Ramirez‐Pena *et al.*, 2010). Putative *rocA* mutant MGAS2221 derivatives were confirmed via PCR and targeted sequencing.

### RNA isolation and quantitative RT‐PCR analysis

Total RNA was isolated from tested GAS strains as previously described (Sumby *et al.*, 2006). Briefly, the strains of interest were grown to the exponential phase (O.D.600 = 0.5) or mid‐stationary phase of growth (3 h after reaching O.D.600 = 0.5) in THY broth. Two volumes of RNAprotect bacteria reagent (Qiagen Inc) were added to one volume of GAS culture and incubated at room temperature for 5 min. Following centrifugation (5,000 *g* for 10 mins at 4°C) the supernatant was discarded, the cell pellets snap frozen in liquid nitrogen, and the frozen pellets placed at −80°C until ready for processing. Cells were processed using a mechanical lysis method with lysing matrix B tubes in conjunction with a FastPrep24 homogenizer (MP Biomedicals). RNA was isolated using the miRNeasy kit (Qiagen) with contaminating DNA being removed with three treatments with TURBO‐DNase‐free (Life Technologies). The quality and quantity of the purified RNA was determined using a Bioanalyzer system (Agilent Tech). Total mRNA samples were converted into cDNA using the reverse transcriptase Superscript III (Life Technologies). The generated cDNA was analyzed via TaqMan‐based quantitative RT‐PCR analysis using a CFX Connect Real‐Time System (Bio‐Rad). TaqMan primers and probes for genes of interest, and the internal control gene *proS*, are shown in Table S2. Transcript levels were determined using the ΔΔ*C_T_* method.

### Creation of MGAS2221 derivatives lacking CovS kinase or phosphatase activity

To assess the requirements of CovS kinase or phosphatase activities to RocA‐mediated regulation we created strains M1covS^Kinase‐KO^ and M1covS^Phos‐KO^. The kinase mutant strain was created via allelic replacement, substituting the wild‐type *covS* gene for an allele expressing an E281A CovS derivative. That substituting an alanine for the glutamate at position 281 results in a CovS protein that lacks kinase activity has been described previously (Horstmann *et al.*, 2015). The phosphatase mutant strain was also created via allelic replacement, substituting the wild‐type *covS* gene for an allele expressing a T284A CovS derivative, a mutation that has also been previously described (Horstmann *et al.*, 2015). Allele replacement was performed in a similar way as described above for the creation of strain M1.RocA‐H246A. Primers used to make the strains are listed in Table S2. Created strains were verified by PCR and targeted sequencing of the *covR*, *covS* and *rocA* genes.

Derivatives of M1covS^Kinase‐KO^ and M1covS^Phos‐KO^ that lack *rocA* were created to enable the analysis of RocA activity in the *covS* mutant strain backgrounds. These strains, M1covS^Kinase‐KO^ΔrocA and M1covS^Phos‐KO^ΔrocA, respectively, were created by replacing the *rocA* gene with a non‐polar spectinomycin resistance cassette, as was previously used to create strain M1ΔrocA (Miller *et al.*, 2015). Replacement of the *rocA* gene with the spectinomycin resistance cassette in the transformants was confirmed via PCR and targeted sequencing.

### Isolation of GAS secreted protein fractions

Aliquots (10 ml) were recovered from GAS strains grown in THY broth to mid‐exponential phase (O.D.600 = 0.5) and mid‐stationary phase (3 h after O.D.600 = 0.5). The cells were pelleted by centrifugation (5,000 × *g* for 20 min at 4°C) and the supernatant filtered through a 0.22‐μm filter into 35 ml of 100% ethanol and precipitated overnight at −20°C. Precipitated proteins were collected by centrifugation (5,000 × *g* for 20 min at 4°C) and resuspended in SDS‐PAGE buffer.

### Isolation of GAS cytoplasmic protein fractions

Cell pellets were gained from GAS strains grown in 100 ml of THY broth to mid‐exponential phase (O.D.600 = 0.5). Cell pellets were resuspended in 500 µl 0.2 M Tris‐HCl buffer (pH 6.8) containing protease inhibitors (Complete Mini Protease Inhibitor Cocktail, Roche) and PhosStop (phosphatase inhibitor cocktail, Roche). Cells were then lysed via mechanical disruption (FastPrep Machine, MP Biomedicals) using speed 5 for 20 s and repeated three times. After centrifugation to pellet the cell debris, 200 µl of the supernatant containing cytoplasmic proteins were removed to a clean tube containing 2X SDS‐PAGE buffer. Protein concentrations were determined by Qubit (Life technologies) according to manufacturer’s recommendations.

### Western blot analyses

Protein samples were separated on 10% SDS‐polyacrylamide gels and transferred to nitrocellulose membranes. The membranes were used in Western blot analyses with custom sheep anti‐SKA polyclonal antibodies (created for us by Pacific Immunology Inc), a commercial rabbit anti‐SLO/SPN polyclonal antibody (American Research Products Inc), a custom rabbit anti‐Spd3 polyclonal antibody (Pacific Immunology Inc) and a commercial horseradish peroxidase (HRP)‐conjugated rabbit anti‐SpeB polyclonal antibody (Toxin Technology) as primary antibodies. The blots were blocked with 5% non ‐fat milk in PBST buffer (2.7 mM potassium chloride, 137 mM sodium chloride pH 7.4 and 0.1% Tween 20) and incubated overnight at 4°C with specific primary antibodies. The proteins were detected using Alexa Fluor 680 donkey anti‐rabbit IgG (at a dilution 1:10,000) or HRP conjugated rabbit anti‐Sheep IgG (Abcam, at a dilution 1:20,000) secondary antibodies. The florescent signal was detected using a Li‐Cor Odyssey Near‐Infrared System or by using a ChemiDoc^TM^ MP Imaging System (BIO‐RAD) in association with the SuperSignal West Femto maximum sensitivity kit (ThermoFisher).

### PhosTag Western blot analysis

Equal amounts (100 µg) of cytoplasmic protein fractions from individual GAS strains were separated on a 10% SDS‐PAGE gel containing 50 μM Phos‐Tag Acrylamide (Wako Pure Chemicals) and 100 μM MnCl_2_ (Sigma‐Aldrich). After electrophoresis, the gel was washed in transfer buffer (SDS running buffer containing 15% MeOH and 10 mM EDTA) twice for 15 min each, and then in transfer buffer without EDTA for 10 min. The separated proteins were transferred to PVDF membrane via wet transfer (400 mA for 45 min). The membrane was used in a standard Western blot procedure using a custom rabbit anti‐CovR antibody, as previously described (Horstmann *et al.*, 2015). The proteins were detected using Alexa Fluor 680 donkey anti‐rabbit IgG (at a dilution 1:10,000) and the florescent signal was detected using a Li‐Cor Odyssey Near‐Infrared System.

### Creation of *stk* mutant GAS derivatives

Strain M1Δ*stk* was created using primers listed in Table S2. Briefly, genomic DNA from the previously created *stk* mutant strain JRS2516 (a gift from Prof. June Scott) (Bugrysheva *et al.*, 2011) was used to PCR amplify the mutant *stk* gene (in which a promoterless spectinomycin resistance gene has been inserted) through use of primers UNR634/637. The PCR product was transformed into MGAS2221 and transformants selected on THY agar plates containing spectinomycin. Insertion of the spectinomycin resistance gene into *stk* in strain M1Δstk was confirmed via PCR and targeted sequencing. To analyze RocA activity in a *stk* mutant background we created strain M1Δ*stk*Δ*rocA* by introducing an in‐frame deletion in *rocA* by allelic replacement in the M1Δstk strain background.

### Fluorescence microscopy

To enable investigation of possible interactions between RocA and CovS via fluorescence microscopy we created strain M1.rocA^FLAG^ pCovS^GFP^, which is a derivative of MGAS2221 in which the chromosomal *rocA* gene has been modified such that the protein has a C‐terminal FLAG‐tag, and which also harbors plasmid pCovS^GFP^ which expresses a CovS protein to which the *gfp* gene from plasmid pOSLA (Poupel *et al.*, 2018) (a gift from Dr. Sarah Dubrac) is fused to the c‐terminus. To make this strain we first created strain M1.rocA^FLAG^ by allelic replacement using suicide vector pBBL740, using a similar approach as described above and previously (Ramirez‐Pena *et al.*, 2010; Miller *et al.*, 2015). Strain M1.rocA^FLAG^ pCovS^GFP^ was created by transforming pCovS^GFP^ into strain M1.rocA^FLAG^. GAS strains were verified by PCR and targeted sequencing.

Fluorescence microscopy was carried out on exponential phase cells grown in THY broth. GAS cells were harvested by centrifugation and re‐suspended in PBS. 10 µL of cells were plated on sterile round coverslips. The cells were air‐dried, fixed with 10% methanol and permeabilized with 0.2% Triton X −100 in PBS for 10 min at room temperature. Cells were blocked with PBS containing 0.4% fish skin gelatin and 0.05% Triton X −100 for 30 min at room temperature. The cells were then incubated with specific primary antibodies [mouse anti‐FLAG (M2, Sigma ‐Aldrich) and rabbit anti‐GFP (Santa Cruz Biotechnology)] overnight at 4ºC and washed with PBS three times before incubating with Alexa Fluor conjugated secondary antibodies (Life Technologies Inc.) for 45 min at room temperature. Cells were visualized and imaged using a confocal laser ‐scanning microscope (Carl 271 Zeiss, Inc.).

### BACTH analyses

Bacterial two‐hybrid assays were performed using GAS proteins fused to the N‐ or C‐termini of either the T18 or T25 portions ofCyaA in plasmids pUT18C/pKT25 (Euromedex) (Karimova *et al.*, 1998). These plasmids were constructed via conventional methods using the primers listed in Table S2. The assay was performed in BTH101 *E. coli* cells co‐transformed with the pKT25 and pUT18C‐based plasmids. Interactions between the target proteins was quantified using β‐galactosidase activity as a measure of re‐constructed CyaA function, in a 96‐well plate format (Battesti and Bouveret, 2012). Briefly, co‐transformed BTH101 cells were grown overnight in 1 ml of rich media (lysogeny broth) (Bertani, 2004) supplemented with kanamycin (50 µg ml^–1^), ampicillin (100 µg ml^–1^) and IPTG (0.5 mM) at 30°C in deep‐well plates. The next morning, 50 µl of cells were transferred to a microplate containing 150 µl of water, and the plate was read at 630 nm to record the optical densities. About 100 µl of the overnight cultures were permeabilized in a polypropylene deep‐well plate by the addition of 20 µl of 0.1% SDS, 40 µl of chloroform and 1 ml of Z‐buffer (60 mM sodium phosphate septahydrate, 10 mM potassium chloride, 40 mM sodium phosphate monohydrate, 1 mM magnesium sulfate septahydrate and 50 mM beta‐mercaptoethanol), followed by aspiration and mixing. Subsequently, 100 µl of the permeabilized cells were transferred to a microplate for the enzymatic reaction. 20 µl of ONPG (4 mg ml^–1^) was added to this solution, and the plate was incubated at room temperature for 1 h, after which time 50 µl of 1M sodium carbonate was added to each well and the plate was read at 415 nm. This activity was then normalized to the cell density and to the level of activity shown for the negate control (empty vector) strain.

### Isolation of GAS membrane protein fractions

Cell pellets were gained from GAS strains grown in 200 ml THY broth to mid‐exponential phase (O.D.600 = 0.5). Cell pellets were resuspended in 6 ml buffer A (0.5 M Tris‐HCl buffer, 2 mM EDTA, 30% sucrose, pH 8.0) supplemented with protease inhibitors (Complete Mini Protease Inhibitor Cocktail). To degrade the peptidoglycan layer, mutanolysin (∼500 units, Sigma) and lysozyme (∼800 µg, Sigma) were added and the samples were incubated at 37°C for 2 h with end‐to‐end rotation. Following incubation, the cells were collected by centrifugation (5,000 × *g* for 10 min at 4°C). The cell pellets were resuspended in 500 µl buffer A and the cells were lysed via mechanical disruption (FastPrep Machine, MP Biomedicals) using speed 5 for 30 s and repeated three times. For further lysis, a Bioruptor cell sonicator (Diagenode) was used (35 cycles for 30 s on and 30 s off). Cell debris was removed by centrifugation (1,000 × *g* for 10 s). The cell membrane fraction was separated from the cytoplasmic fraction by ultracentrifugation of the supernatants for 1 h at 90,000 × *g* at 4°C (Beckman Coulter). Cell pellets were resuspended in 1 ml buffer B (50 mM sodium phosphate, 300 mM NaCl) and 0.95% Triton X‐100 was added to each sample. The cell suspensions were incubated at room temperature for 10 min with end‐to‐end rotation, to obtain the membrane fraction.

### Co‐immunoprecipitation

To further facilitate analysis of putative interactions between RocA and CovS we created two additional GAS strain backgrounds, M1.CovS^HIS^ and M1.FasB^HIS^ (Table S1). Strains M1.CovS^HIS^ and M1.FasB^HIS^ express chromosomally encoded, C‐terminal HIS‐tagged CovS or FasB proteins, respectively. The two new strain backgrounds were created using a similar approach as described above for strain M1.RocA‐H246A. A series of double‐tagged derivative strains were also created via the introduction of the pDC123‐based plasmid pRocA^FLAG^, which expresses a C‐terminal FLAG‐tagged RocA protein.

To perform co‐immunoprecipitation, membrane protein fractions of the desired GAS strains were mixed with 1X lysis buffer (50 mM Tris‐HCl buffer pH 8, 150 mM NaCl, 1 mM EDTA, 1% Triton X‐100) in a 1:1 ratio. About 500 µg of each protein sample were incubated with specific antibody‐coated beads [Anti‐FLAG M2 magnetic beads (Sigma Aldrich) or anti‐HIS mouse mAb (Cell Signaling)] overnight at 4°C. The bead‐bound immunocomplexes were collected using a magnetic bead concentrator (DynaMag^TM^, Invitrogen). The beads were washed with either 1X TBS (50 mM Tris‐HCl buffer pH 8, 150 mM NaCl, for FLAG beads) or 1X lysis buffer (for HIS beads) five times. The beads were boiled in 25 µl 2X‐SDS PAGE buffer for 5 min, the proteins were recovered and quantified, and equal concentrations of each were resolved on SDS‐polyacrylamide gels. Western blot analysis was carried out using a specific rabbit anti‐FLAG antibody (Cell Signaling). The FLAG‐tagged proteins were detected following incubation with Alexa Fluor 680 donkey anti‐rabbit IgG (at a dilution 1:10,000) secondary antibody and the florescent signal was detected using a Li‐Cor Odyssey Near‐Infrared System.

### RNA‐Seq analyses

The M1 and M3 GAS strains tested were grown to the stationary phase of growth (4 h after reaching an OD_600_ of 0.5) in THY broth. Total RNA was isolated and rRNAs were depleted using the Ribo‐Zero Gram‐positive rRNA removal kit (Illumina, Inc.). The rRNA‐depleted RNA was then used to generate cDNA libraries for sequencing using the ScriptSeq V2 kit (Illumina, Inc.). Briefly, RNA was fragmented, cDNA was synthesized using random hexamers containing a 5′ tagging sequence, RNA was hydrolyzed, and the cDNA was tagged at the 3′ end. A limited number of PCR cycles (*n* = 12 to 14) were used to amplify the libraries via the 5′ and 3′ tags (the libraries were barcoded using different primers), and the libraries were size selected (170 to 300 bp). The size‐selected and barcoded libraries were run on an Illumina flow cell using an Illumina HiSeq2000 instrument. Data were analyzed using CLC Genomics Workbench and normalized to the overall sequencing depth using total mapped read data. Statistical significance was tested using Kal's Z test with a false‐discovery rate correction. The RNAseq data have been deposited at the Gene Expression Omnibus (GEO) database at the National Center for Biotechnology Information (http://www.ncbi.nlm.nih.gov/geo) and are accessible through accession numbers http://www.ncbi.nlm.nih.gov/geo/query/acc.cgi?acc=GSE131235 and http://www.ncbi.nlm.nih.gov/geo/query/acc.cgi?acc=GSE131239.

### Creation of a spectinomycin‐resistant *covS* deletion mutant strain

A *covS* deletion mutant derivative (2221ΔcovS::Spec) of parental M1 strain MGAS2221 was created using the primers listed in Table S2. Briefly, 1kb regions upstream and downstream of M1 *covS* gene were amplified along with the non‐polar spectinomycin resistance cassette from plasmid pSL60 (Lukomski *et al.*, 2000). The PCR products were joined together via overlap PCR, and the resultant 3 kb PCR product was cloned and sequenced to ensure no spurious mutations had been introduced during the PCR process. Subsequently, the 3 kb insert was PCR amplified and transformed into the parental serotype M1 strain MGAS2221. Replacement of the *covS* gene with the spectinomycin resistance cassette was confirmed via PCR and targeted sequencing.

### Saliva growth competition assays

Six competition assays were performed following the protocol that we described previously (Trevino *et al.*, 2009). These assays consisted of all possible combinations of strain pairs from four different strains: the parental isolate MGAS2221 and the isogenic derivatives 2221ΔcovR, 2221ΔcovS::Spec and 2221ΔrocA (see Table S1 for more information). Briefly, 100 µl of overnight THY broth cultures of the two strains to be compared were diluted 1:100 using 9.9 ml of sterile PBS. The two strains were mixed together and further diluted by adding 100 μl of each of the PBS‐diluted GAS to 9.8 ml of human saliva. After vortexing to ensure homogeneity, a 100‐μl sample was removed, serially diluted and plated on blood agar plates (two plates per dilution; this was to enable calculation of the initial ratio of each strain). The remainder of the GAS‐inoculated saliva was incubated at 37°C for 12 h. After incubation, a 100‐μl sample was removed, serially diluted and plated on blood agar plates as described above. In addition, a 5‐μl sample was inoculated into 4.995 ml of fresh human saliva, the preparation was incubated for 12 h at 37°C, a 100‐μl aliquot was removed, the titer was determined, and 5 μl was inoculated into 4.995 ml of fresh saliva. This cycle was repeated for a total of 72 h (six saliva aliquots). The number of CFU of each strain in the saliva was determined using a combination of colony morphology on blood agar plates (MGAS2221 produces small, non‐mucoid colonies, while the *covR*, *covS* and *rocA* mutant strains produce larger, mucoid colonies) and antibiotic resistance profiling (by patching colonies obtained from blood agar plates onto THY agar plates containing kanamycin or spectinomycin or both).
