# Supplementary material for: The group A Streptococcus accessory protein RocA: regulatory activity, interacting partners and influence on disease potential
Source: Mol Microbiol. 2019 Nov 11;113(1):190–207. doi: 10.1111/mmi.14410 (PMC7028121; doi:10.1111/mmi.14410)
Supplement: Supplementary file 1 [file MMI-113-190-s001.pdf]

Strain	Description	Reference
M1	Parental M1 GAS strain MGAS2221 (*covRS*$^{+}$ *rocA*$^{+}$)	Sumby *et al*., 2006. *PLoS Pathog* **2**:e5
M1ΔrocA	MGAS2221 derivative in which *rocA* has been replaced with a spectinomycin resistance cassette	Miller *et al*., 2015. *Mol Microbiol* **98**:473-89
M1.RocA-H246A	MGAS2221 derivative in which *rocA* has been replaced with an allele that results in an H246A substitution	This work
M1ΔcovS	MGAS2221 derivative that contains a mutant *covS* allele due to the deletion of a single nucleotide	Sumby *et al*., 2006. *PLoS Pathog* **2**:e5
M1covS$^{Kinase\text{-}KO}$	MGAS2221 derivative that, due to an E281A amino acid substitution, produces a kinase-deficent CovS protein	This work
M1covS$^{Kinase\text{-}KO}$ΔrocA	A derivative to M1covS$^{Kinase\text{-}KO}$ in which the rocA gene has been replaced with a spectinomycin resistance cassette	This work
M1covS$^{Phos\text{-}KO}$	MGAS2221 derivative that, due to an E284A amino acid substitution, produces a phosphatase-deficent CovS protein	This work
M1covS$^{Phos\text{-}KO}$ΔrocA	A derivative to M1covS$^{Phos\text{-}KO}$ in which the rocA gene has been replaced with a spectinomycin resistance cassette	This work
M1ΔcovR	MGAS2221 derivative in which the region of *covR* that encodes the DNA-binding domain has been replaced with a kanamycin resistance cassette	Trevino *et al*., 2009. *Infect Immun* **77**:3141-9
M1Δstk	MGAS2221 derivative in which *stk* has been replaced with a spectinomycin resistance cassette	This work
M1ΔstkΔrocA	M1Δstk derivative in which *rocA* has been mutated through the introduction of a premature stop codon	This work
M1.rocA^{FLAG}	MGAS2221 derivative that has a chromosomally expressed RocA harboring a C-terminal FLAG-tag	This work
M1.rocA^{FLAG} pCovS-GFP	Derivative of M1.rocA^{FLAG} that contains plasmid pCovS-GFP which expresses a CovS-GFP fusion protein	This work
M1.covS^{HIS}	MGAS2221 derivative that has a chromosomally expressed CovS harboring a C-terminal HIS-tag	This work
M1 pRocA^{FLAG}	Derivative of MGAS2221 that contains plasmid pRocA^{FLAG} which expresses RocA with a C-terminal FLAG-tag	This work
M1.covS^{HIS} pRocA^{FLAG}	Derivative of M1.covS^{HIS} that contains plasmid pRocA^{FLAG} which expresses RocA with a C-terminal FLAG-tag	This work
M1.fasB^{HIS}	MGAS2221 derivative that has a chromosomally-expressed FasB harboring a C-terminal HIS-tag	This work
M1.fasB^{HIS} pRocA^{FLAG}	Derivative of M1.fasB^{HIS} that contains plasmid pRocA^{FLAG} which expresses RocA with a C-terminal FLAG-tag	This work
M1ΔrocA^{Comp}	Complemented derivative of M1ΔrocA in which the spectinomycin resistance cassette has been replaced with a functional *rocA* allele	Miller *et al*., 2015. *Mol Microbiol* **98**:473-89
M3	Parental M3 GAS strain MGAS10870 (*covRS*$^{+}$ *rocA*$^{-}$)	Beres *et al*., 2004. *Proc Natl Acad Sci USA* **101**:11833-8
M3rocA^{COMP}	MGAS10870 derivative in which the natural mutant *rocA* allele has been replaced with a functional allele from M1 GAS	Miller *et al*., 2015. *Mol Microbiol* **98**:473-89
M3rocA^{MUT}	Derivative of M3rocA^{COMP} in which the functional *rocA* allele has been reverted back into the mutant (M3-like) allele	Miller *et al*., 2015. *Mol Microbiol* **98**:473-89
M1ΔcovS::Spec	MGAS2221 derivative in which *covS* has been replaced with a spectinomycin resistance cassette	This work
M1 + vector	MGAS2221 derivative containing the empty shuttle vector pDCBB	Ramalinga *et al*., 2016. *J Bacteriol* **199**:e00452-16.
M1 + pCovS	MGAS2221 derivative containing the pDCBB-based plasmid pCovS, which expresses a full-length CovS protein	This work
M1 + pRocA	MGAS2221 derivative containing the pDCBB-based plasmid pRocA, which expresses a full-length RocA protein	This work
M1ΔrocA + vector	Derivative of M1ΔrocA that contains the empty shuttle vector pDCBB	This work
M1ΔrocA + pCovS	Derivative of M1ΔrocA that contains the pDCBB-based plasmid pCovS, which expresses a full-length CovS protein	This work

Table S1
GAS strains used in this study.

**Primer name**	**Sequence**	**Purpose**
UNR798	TGTACAATTGCTAGCGTACGGCCgttattaccacacaaacaaaccaacac	Used with UNR802 to amplify a flanking region that was used in the construction of a plasmid to convert H246 of RocA into A246
UNR800	gtgttggtttgtttgtgtggtaataacGGCCGTACGCTAGCAATTGTACA	Used with UNR803 to amplify pBBL740 for use in making a plasmid to convert H246 of RocA into A246
UNR801	GAATATCTTGATACAGCAATCCAAGtgcCTGACTATAATTAATCAGCTCACGATATTC	Used with UNR804 to amplify a flanking region that was used in the construction of a plasmid to convert H246 of RocA into A246
UNR802	GAATATCGTGAGCTGATTAATTATAGTCAGgcaCTTGGATTGCTGTATCAAGATATTC	Used with UNR798 to amplify a flanking region that was used in the construction of a plasmid to convert H246 of RocA into A246
UNR803	GCATTTATCTTTAATATCTAACACATAAAGACTCACgaactagagcttgatgaaaatttgtttg	Used with UNR800 to amplify pBBL740 for use in making a plasmid to convert H246 of RocA into A246
UNR804	caaacaaattttcatcaagctctagttcGTGAGTCTTTATGTGTTAGATATTAAAGATAAATGC	Used with UNR801 to amplify a flanking region that was used in the construction of a plasmid to convert H246 of RocA into A246
UNR858	CGAAAAACAAACAGAGGTTGCATGAAAAAGAGTCTGGGACTATTGTCTCAG	Used with UNR861 to amplify a FLAG-tagged version of *rocA*
UNR859	CTGAGACAATAGTCCCAGACTCTTTTTCATGCAACCTCTGTTTGTTTTTCG	Used with UNR860 to amplify pDC123 for use in cloning a *rocA* gene that expresses a C-terminally FLAG-tagged RocA protein
UNR860	CTCAGATTTATCAGTTGTTGATCTCATCAGAACTTAAGGCGATTAAGCCAATCG	Used with UNR859 to amplify pDC123 for use in cloning a *rocA* gene that expresses a C-terminally FLAG-tagged RocA protein
UNR861	CGATTGGCTTAATCGCCTTAAGTTCTGATGAGATCAACAACTGATAAATCTGAG	Used with UNR858 to amplify a FLAG-tagged version of *rocA*
UNR862	GTTGTACAAATTTATCTGAACAGTTATTCGTGAGAAATAAGTCATATGGAAAATC	Used with UNR865 to amplify a HIS-tagged *covS* gene
UNR863	GATTTTCCATATGACTTATTTCTCACGAATAACTGTTCAGATAAATTTGTACAAC	Used with UNR864 to amplify pDC123 for use in cloning a *covS* gene that will express a C-terminally HIS-tagged CovS protein
UNR864	GCAATGATGGATGACATGAAAGAAATACTCAGAACTTAAGGCGATTAAGCCAATCG	Used with UNR863 to amplify pDC123 for use in cloning a *covS* gene that will express a C-terminally HIS-tagged CovS protein
UNR865	CGATTGGCTTAATCGCCTTAAGTTCTGAGTATTTCTTTCATGTCATCCATCATTGC	Used with UNR862 to amplify a HIS-tagged *covS* gene
UNR946	CACAAATACTCTAGATGCGAATCAAGaatacatgttataataactataactaataacg	Used with UNR949 to amplify the SPEC cassette for use in making a *covS* mutant strain derivative of MGAS2221
UNR947	CTATTTAAATAACAGATTAAAAAAATTATAAgaacggataaatcacgtaatcgaacaag	Used with UNR951 to amplify a ~900bp flank of *covS* for use in making a *covS* mutant strain
UNR948	cgttattagttatagttattataacatgtattCTTGATTCGCATCTAGAGTATTTGTG	Used with UNR950 to amplify a ~900bp flank of *covS* for use in making a *covS* mutant strain
UNR949	cttgttcgattacgtgatttatccgttcTTATAATTTTTTTAATCTGTTATTTAAATAG	Used with UNR946 to amplify the SPEC cassette for use in making a *covS* mutant strain derivative of MGAS2221
UNR950	TCGTGAAGGGTTAGAAACTGCTTTG	Used with UNR948 to amplify a ~900bp flank of *covS* for use in making a *covS* mutant strain
UNR951	GTACGCGATTCGTAATTTGTTGC	Used with UNR947 to amplify a ~900bp flank of *covS* for use in making a *covS* mutant strain
UNR447	CCATGACACAAAAGGTATATTCTAATC	Used with UNR449 to amplify part of *covS* for use in placing a HIS-tag at the C-term
UNR448	CCTTTGGCCCAGTCTAAAGAGAGTcatcaccatcaccatcatTAGAGTAGCTTAAGGCATTGTTAATGTGAC	Used with UNR450 to amplify part of *covS* for use in placing a HIS-tag at the C-term
UNR449	GTCACATTAACAATGCCTTAAGCTACTCTAatgatggtgatggtgatgACTCTCTTTAGACTGGGCCAAAGG	Used with UNR447 to amplify part of *covS* for use in placing a HIS-tag at the C-term
UNR450	GAATAAGCTGGATGTTGAAAAAACAAGTC	Used with UNR448 to amplify part of *covS* for use in placing a HIS-tag at the C-term
UNR451	GACTTGTTTTTTCAACATCCAGCTTATTCgaactagagcttgatgaaaatttgtttg	Used with UNR452 to amplify pBBL740 and use it to place a HIS-tag at the C-term of *covS*
UNR452	GATTAGAATATACCTTTTGTGTCATGGcgtacgctagcaattgtaca	Used with UNR451 to amplify pBBL740 and use it to place a HIS-tag at the C-term of *covS*
UNR33	GCCATTCCTATCTCCGCAGATAAGCTC	Used with UNR455 to amplify part of *rocA* for use in placing a FLAG-tag at the C-term
UNR455	CAGTTAATAGAAATAGCTAAGCCTGACgattataaagatgacgatgataaaTGATGTTAAAGGTATGAATAATCACTC	Used with UNR33 to amplify part of *rocA* for use in placing a FLAG-tag at the C-term
UNR456	GAGTGATTATTCATACCTTTAACATCAtttatcatcgtcatctttataatcGTCAGGCTTAGCTATTTCTATTAACTG	Used with UNR457 to amplify part of *rocA* for use in placing a FLAG-tag at the C-term
UNR457	GCCATTGTTTGGTATGCCTTATTCAG	Used with UNR456 to amplify part of *rocA* for use in placing a FLAG-tag at the C-term
UNR458	CTGAATAAGGCATACCAAACAATGGCgaactagagcttgatgaaaatttgtttg	Used with UNR459 to amplify pBBL740 and use it to place a FLAG-tag at the C-term of *rocA*
UNR459	GAGCTTATCTGCGGAGATAGGAATGGCcgtacgctagcaattgtaca	Used with UNR458 to amplify pBBL740 and use it to place a FLAG-tag at the C-term of *rocA*
UNR549	CACGTTTTATTAGTGATGTCAGCCATGcATTACGAACACCGGTTGCGATTATTAAAG	Use with UNR557 to amplify a section of *covS* for use in creating a CovS-E281A derivative
UNR550	CTTTAATAATCGCAACCGGTGTTCGTAATgCATGGCTGACATCACTAATAAAACGTG	Use with UNR558 to amplify a section of *covS* for use in creating a CovS-E281A derivative
UNR551	ATTAGTGATGTCAGCCATGAATTACGAgCACCGGTTGCGATTATTAAAGGGCATATTGG	Use with UNR557 to amplify a section of *covS* for use in creating a CovS-T284A derivative
UNR552	CCAATATGCCCTTTAATAATCGCAACCGGTGcTCGTAATTCATGGCTGACATCACTAAT	Use with UNR558 to amplify a section of *covS* for use in creating a CovS-T284A derivative
UNR556	GCTTTACTACCTTTGAGCGTGTgaactagagcttgatgaaaatttgtttg	Use with UNR559 to amplify pBBL740 for use in creating CovS kinase or phosphatase mutant derivatives
UNR557	caaacaaattttcatcaagctctagttcACACGCTCAAAGGTAGTAAAGC	Use with UNR549 or UNR551.
UNR558	TGTACAATTGCTAGCGTACGgtcaaatgtttggaaatatgatgaag	Use with UNR550 or UNR552.
UNR32	CCAGCCAAATCATGACCAACC	Use with UNR39 to amplify M1Δ*rocA* strain for creating *rocA* mutant derivative of strains M1CovSE281-A and M1CovS T284-A
UNR39	GTGGGGTTGGCTCGATTGGCTTTGC	Use with UNR32 to amplify M1Δ*rocA* strain for creating *rocA* mutant derivative of strains M1CovSE281-A and M1CovS T284-A
UNR559	cttcatcatatttccaaacatttgacCGTACGCTAGCAATTGTACA	Use with UNR556 to amplify pBBL740 for use in creating CovS kinase or phosphatase mutant derivatives
UNR634	GTGTTTGTCAAACCTTGCGTAAAGGTGG	Use with UNR637 to amplify *stk* mutant strain JRS2516 for creation of strain M1Δ*stk* (deletion mutant derivative of MGAS2221)
UNR637	CGCTTACTGTTATCATCCATAATAATCC	Use with UNR634 to amplify *stk* mutant strain JRS2516 for creation of strain M1Δ*stk* (deletion mutant derivative of MGAS2221)
UNR527	CAATAAAAGCTAAGTTTTAAATGTTTTATGCCTTTTTTCCACTAGTGTC	Use with UNR 732 to amplify ~1000bp flank of *rocA* to create *rocA* mutant derivative of strain M1Δ*stk*
UNR528	GCATAAAACATTTAAAACTTAGCTTTTATTGATTTACCATAGTATAGG	Use with UNR 734 to amplify ~1000bp flank of *rocA* to create *rocA* mutant derivative of strain M1Δ*stk*
UNR732	TGTACAATTGCTAGCGTACGGCCggtcgtgacattccttaagtcaaattg	Use with UNR 527 to amplify ~1000bp flank of *rocA* to create *rocA* mutant derivative of strain M1Δ*stk*
UNR734	caaacaaattttcatcaagctctagttcGTATGATTGAGTGGCAATAACAACTCAAG	Use with UNR 528 to amplify ~1000bp flank of *rocA* to create *rocA* mutant derivative of strain M1Δ*stk*
UNR733	caatttgacttaaggaatgtcacgaccGGCCGTACGCTAGCAATTGTACA	Use with UNR735 to amplify pBBL740 for use in creating rocA mutant derivative of strain M1Δstk
UNR735	CTTGAGTTGTTATTGCCACTCAATCATACgaactagagcttgatgaaaatttgtttg	Use with UNR733 to amplify pBBL740 for use in creating rocA mutant derivative of strain M1Δstk
UNR907	CTGTAAATAATTCTTCTCCTTTTGACCCTGACCCTGACCCGGGACTCTCTTTAGACTGGGCCAAAGGAATATGTAAGATAAACAC	Use with UNR 908 to amplify the plasmid backbone and *covS* gene from pCovS-His for creating pCovS-GFP
UNR908	GGAATTACACACGGAATGGATGAATTATATAAGTAAAGTAGCTTAAGGCATTGTTAATGTGACAGGTATAG	Use with UNR 907 to amplify the plasmid backbone and *covS* gene from pCovS-His for creating pCovSGFP
UNR910	GATGGCATTTTAACCCAGTTAATAGAAATAGCTAAGCCTGACCCCGGGTCAGGGTCAGGGTCAAAAGGAGAAGAATTATTTACAG	Use with UNR 913 to amplify GFP from pOSLA for creating pCovSGFP
UNR913	CAGAGGTTGCATGAAAAAGAGTCTGGGACTATTGTCTCAGTTACTTATATAATTCATCCATTCCGTGTGTAATTCC	Use with UNR 913 to amplify GFP from pOSLA for creating pCovSGFP
UNR560	TGCTCACATGTTCTTTCCTGC	Use with UNR561 to amplify inserts from plasmids pUT18 and pUT18C
UNR561	GGTGAAAACCTCTGACACATGC	Use with UNR560 to amplify inserts from plasmids pUT18 and pUT18C
UNR562	CACAGTTAAATTGCTAACGCAGTC	Use with UNR563 to amplify inserts from plasmid pKT25
UNR563	ATCTCATGCTGGAGTTCTTCG	Use with UNR562 to amplify inserts from plasmids pKT25 and pKNT25
UNR564	GAGGAAGCGGAATATATCCTG	Use with UNR563 to amplify inserts from plasmid pKNT25
UNR565	GAATCTTTCCAAACTGTTCGG	Primer to help sequence T25
UNR566	TTGGACAGATGCGGCATAATG	Primer to help sequence T25
UNR567	ATGCGATTGCTGCATGGTCATTG	Primer to help sequence T25
UNR568	GCACCGTTTCCAGTACATC	Primer to help sequence T18
UNR569	ATTTCGAGCTGGAAGTGCGCAATG	Primer to help sequence T18
UNR570	GACTGAATTCgaTTTCTCACGAATAACGTATCCC	Use with UNR572 for CovR C-term (no STOP codon, EcoRI, for pKNT25 / pUT18)
UNR571	GACTGAATTCTTATTTCTCACGAATAACGTATCCC	Use with UNR572 CovR C-term (WITH STOP codon, EcoRI, for pKT25 / pUT18C)
UNR572	GACTGGTACCGACAAAGAAAATTTTAATTATTGAAGATG	Use with UNR 570 or UNR 571 CovR N-term (no START codon, KpnI)
UNR573	GACTGAATTCCTAACTCTCTTTAGACTGGGCC	Use with UNR 575 CovS C-term (WITH STOP codon, EcoRI, for pKT25 / pUT18C)
UNR574	GACTGAATTCgaACTCTCTTTAGACTGGGCC	Use with UNR575CovS C-term (no STOP codon, EcoRI, for pKNT25 / pUT18)
UNR575	GACTGGTACCGGAAAATCAGAAACAAAAACAG	Use with UNR 574 or UNR 573 CovS N-term (no START codon, KpnI)
UNR576	GACTGAATTCTCAGTCAGGCTTAGCTATTTCTATTAACTG	Use with UNR 578 RocA C-term (WITH STOP codon, EcoRI, for pKT25 / pUT18C)
UNR577	GACTGAATTCgaGTCAGGCTTAGCTATTTCTATTAACTG	Use with UNR 578 RocA C-term (no STOP codon, EcoRI, for pKNT25 / pUT18)
UNR578	GACTGGTACCGTTAGAAGATTTTCTTCAATTTTTAGG	Use with UNR 577 or UNR 576 RocA N-term (no START codon, KpnI)
proS.UTMF	TACCACTGGCAAATCGTACC	Taqman primer for the house-keeping gene *proS*
proS.UTMR	CATTTCAACAGCACCGATCT	Taqman primer for the house-keeping gene *proS*
proS.UTMP	CACGCATGATGGTCTTGAATTTCTCA	Taqman probe for the house-keeping gene *proS*
rocATMF	AGGGCTATAAGCGCAAAGAA	Taqman primer for the gene *rocA*
rocATMR	GGCTTTCTTTCCAGACCATT	Taqman primer for the gene *rocA*
rocATMP	TGAGCCAACATCACAACATCAAGAATG	Taqman probe for the gene *rocA*
hasATMF	ATGATCGATGTTTAACAAATTATGCTATTG	Taqman primer for the gene *hasA*
hasATMR	TTAAATAACTTTTTAATTGGAAAGGTACATCAG	Taqman primer for the gene *hasA*
hasATMP	ACGCACTGTCTACCAATCAACAGCTAGATGTG	Taqman probe for the gene *hasA*
scpCTMF	AAGGAGCTTGGGACAAGGGATA	Taqman primer for the gene *scpC*
scpCTMR	TGATGGGCCGGATCGA	Taqman primer for the gene *scpC*
scpCTMP	CAATAACTGCGACAACCTTGCCTTGTCCT	Taqman probe for the gene *scpC*
sloTMF	GACCTTTAAAGAGTTGCAACGAAAA	Taqman primer for the gene *slo*
sloTMR	GACCATAAGCTACGTTACTCACAAAGA	Taqman primer for the gene *slo*
sloTMP	TGTCAGCAATGAAGCCCCGCC	Taqman probe for the gene *slo*
grabTMF	GCATCAGTATTAGTCGGTTCAACAGT	Taqman primer for the gene *grab*
grabTMR	GGTTCCGCCATTTGGAATAA	Taqman primer for the gene *grab*
grabTMP	TGTTGACTCACCTATCGAACAGCCTCGA	Taqman probe for the gene *grab*
skaTMF	CGGCTACTTTGAGGTCATTGATT	Taqman primer for the gene *ska*
skaTMR	CCGAACCATCTTTGTCAGCAA	Taqman primer for the gene *ska*
skaTMP	CAAGCGATGCAACCATTACTGATCGAAAC	Taqman probe for the gene *ska*
speBTMF	TGCAGGTAGCTCTCGTGTTCA	Taqman primer for the gene *speB*
speBTMR	TGCTTCCCAATCTTGTTTGCT	Taqman primer for the gene *speB*
speBTMP	ATCTGTTCACCAAATCAACCGTGGCG	Taqman probe for the gene *speB*
sagCTMF	CTGCTGAGTTGCTAGCTGAACAAC	Taqman primer for the gene *sagC*
sagCTMR	cttTGGTAATCGCACAAGGCATCtg	Taqman primer for the gene *sagC*
sagCTMP	CGGCGATGCTCCAAAGCATCTAAACGCgaac	Taqman probe for the gene *sagC*

Table S2
Primers and probes used in this study

Gene name	Fold change in mRNA abundance
M5005_Spy0667	10.500
spd3	8.120
M5005_Spy0125	7.126
M5005_Spy1170	6.349
sdaD2	6.214
M5005_Spy0123	5.549
ntpI	5.511
ntpF	5.420
ntpC	5.080
nga	4.786
arcA	4.772
ntpA	4.553
ntpE	4.388
ntpB	4.362
ntpD	4.317
ntpK	4.310
sclA	4.079
M5005_Spy0140	4.041
sagC	3.984
M5005_Spy1619	3.769
sagD	3.757
sagI	3.639
slo	3.639
M5005_Spy1407	3.633
sloR	3.524
sagE	3.490
sagH	3.462
sic1.01	3.342
sagB	3.308
sagF	3.268
sagA	3.229
sagG	3.209
emm1.0	3.180
M5005_Spy0920	3.011
M5005_Spy0247	2.768
M5005_Spy1291	2.705
M5005_Spy1190	2.667
M5005_Spy0861	2.652
M5005_Spy0255	2.643
M5005_Spy1089	2.597
dppC	2.593
M5005_Spy1174	2.586
dppB	2.552
M5005_Spy1759	2.521
sdhB	2.471
hasA	2.462
M5005_Spy1239	2.450
rpmG	2.439
M5005_Spy1290	2.405
M5005_Spy1760	2.400
M5005_Spy0095	2.391
rpmF	2.387
rpsT	2.378
czcD	2.349
M5005_Spy1766	2.316
hasB	2.287
sdhA	2.277
M5005_Spy1020	2.275
rpmH	2.269
M5005_Spy0102	2.261
rpmB	2.234
M5005_Spy0121	2.224
M5005_Spy1579	2.223
M5005_Spy1086	2.187
M5005_Spy1078	2.185
M5005_Spy1289	2.179
dppE	2.136
M5005_Spy0652	2.129
M5005_Spy1312	2.108
M5005_Spy0118	2.107
M5005_Spy0571	2.088
dppA	2.084
M5005_Spy1850	2.070
M5005_Spy1663	2.064
M5005_Spy0597	2.061
rpsN2	2.058
M5005_Spy0003	2.053
M5005_Spy0442	2.047
M5005_Spy1666	2.037
M5005_Spy0488	2.037
M5005_Spy0399	2.031
scpA	2.029
coaA	2.023

M5005_Spy1505	2.019
M5005_Spy1286	2.003
ccdA	-0.500
M5005_Spy1556	-0.500
M5005_Spy0311	-2.015
M5005_Spy0032	-2.026
M5005_Spy1006	-2.029
M5005_Spy1449	-2.043
M5005_Spy1417	-2.057
M5005_Spy0914	-2.060
M5005_Spy0984	-2.077
M5005_Spy0871	-2.079
M5005_Spy0500	-2.081
M5005_Spy0777	-2.102
M5005_Spy1460	-2.125
M5005_Spy1443	-2.163
M5005_Spy0870	-2.163
M5005_Spy1411	-2.176
M5005_Spy1749	-2.178
M5005_Spy0718	-2.186
M5005_Spy1442	-2.206
M5005_Spy1441	-2.216
M5005_Spy1422	-2.223
M5005_Spy0676	-2.231
M5005_Spy1205	-2.233
pyrB	-2.249
M5005_Spy1433	-2.257
M5005_Spy1202	-2.267
glyA	-2.276
M5005_Spy1432	-2.282
M5005_Spy1416	-2.288
M5005_Spy0165	-2.288
M5005_Spy0982	-2.294
M5005_Spy1201	-2.302
M5005_Spy1859	-2.316
M5005_Spy1450	-2.321
rbfA	-2.330
M5005_Spy1448	-2.333
M5005_Spy1437	-2.344
comYA	-2.348
carA	-2.362
mur1.1	-2.370
M5005_Spy0866	-2.380
M5005_Spy1430	-2.398
M5005_Spy1424	-2.398
M5005_Spy1426	-2.454
M5005_Spy1423	-2.502
M5005_Spy1787	-2.523
M5005_Spy1425	-2.543
M5005_Spy1421	-2.569
dnaJ	-2.583
M5005_Spy1828	-2.641
M5005_Spy1447	-2.643
M5005_Spy1438	-2.667
M5005_Spy1732	-2.682
M5005_Spy1444	-2.705
M5005_Spy1440	-2.760
isp	-2.766
M5005_Spy1428	-2.768
M5005_Spy1668	-2.833
M5005_Spy1786	-2.835
M5005_Spy1218	-2.857
ihk	-2.866
M5005_Spy0114	-2.876
xerD	-2.982
irr	-2.984
M5005_Spy1750	-3.000
M5005_Spy1429	-3.000
M5005_Spy0719	-3.010
M5005_Spy1435	-3.022
M5005_Spy1862	-3.030
grab	-3.041
M5005_Spy1722	-3.099
M5005_Spy1419	-3.106
carB	-3.126
M5005_Spy1456	-3.238
M5005_Spy1434	-3.259
M5005_Spy1181	-3.273
M5005_Spy1439	-3.278
M5005_Spy1729	-3.344
M5005_Spy0169	-3.412
M5005_Spy0859	-3.493
scrA	-3.499
M5005_Spy1214	-3.532

endoS	-3.558
speB	-3.605
M5005_Spy1414	-3.684
smf	-3.833
fruB	-3.865
M5005_Spy1860	-3.979
M5005_Spy1207	-4.000
M5005_Spy1726	-4.043
M5005_Spy1477	-4.194
M5005_Spy1728	-4.466
xpt	-4.525
M5005_Spy0186	-4.617
fruA	-4.711
M5005_Spy1736	-4.745
fruR	-4.861
M5005_Spy1727	-4.959
M5005_Spy0034	-5.059
M5005_Spy1187	-5.167
purF	-5.205
M5005_Spy1733	-5.236
M5005_Spy0022	-5.397
purM	-5.451
purN	-5.746
M5005_Spy0027	-5.987
scrK	-6.734
M5005_Spy1827	-7.850
M5005_Spy0023	-7.882
spi_2	-7.898
M5005_Spy1826	-8.978
M5005_Spy0090	-9.333
purE	-9.412
M5005_Spy1825	-9.430
purK	-10.035
purD	-10.403
M5005_Spy0028	-11.563
M5005_Spy1667	-13.000
opuABC	-13.578
opuAA	-14.210
rocA	-51.460

Table S3

Genes differentially regulated between our parental M1 isolate and its derivative M1ΔrocA. Color-coding is consistent with that shown in figure 8A.

Gene name	Fold change in mRNA abundance
SpyM3_1680	47.604
SpyM3_1679	43.750
SpyM3_1677	42.512
SpyM3_1678	38.628
SpyM3_1775	38.615
SpyM3_1777	38.573
hutH	30.903
sagE	27.112
fhs.2	26.489
SpyM3_0181	23.690
SpyM3_1774	19.767
sagA	19.016
sagF	18.864
sagC	18.718
SpyM3_1291	18.665
sagG	18.621
SpyM3_1290	18.257
SpyM3_1778	18.193
sagH	17.601
sagB	17.231
SpyM3_1289	16.315
SpyM3_0186	15.393
SpyM3_1292	15.373
SpyM3_1001	15.275
hutU	15.225
sagD	15.150
lacA.1	15.059
sagI	14.964
SpyM3_1487	14.755
lacD.1	14.446
lacB.1	14.413
dexB	14.385
SpyM3_0180	14.302
SpyM3_0183	14.270
SpyM3_0179	14.207
SpyM3_0182	13.920
rgfB	13.771
hutI	13.712
SpyM3_1488	13.514
SpyM3_1288	13.252
SpyM3_1002	13.100
pulA	12.969
lacC.1	12.768
nanH	12.281
SpyM3_1000	11.645
malE	10.816
SpyM3_0184	10.763
SpyM3_1708	10.277
SpyM3_1287	10.185
sla	10.184
SpyM3_1486	9.993
SpyM3_1286	9.905
SpyM3_1781	9.721
salA	9.613
SpyM3_1005	9.483
malG	9.369
dexS	9.368
SpyM3_0771	9.347
SpyM3_1285	9.300
SpyM3_1003	8.576
SpyM3_0772	8.554
npx	8.519
nra	8.009
malP	7.998
sclA_2	7.960
mf	7.677
arcC	7.575
SpyM3_1004	7.553
salX	7.284
SpyM3_1763	7.158
SpyM3_1295	7.082
SpyM3_0304	6.947
malF	6.890
SpyM3_1650	6.753
SpyM3_1006	6.588
salT	6.501
bglA.2	6.352
SpyM3_1293	6.309
SpyM3_0773	6.206
salB	6.183
agaF	6.147
ntpA	5.999

copZ	5.861
SpyM3_1296	5.778
ntpC	5.750
SpyM3_1192	5.678
SpyM3_1464	5.623
SpyM3_1119	5.562
spyCEP	5.447
lacR.1	5.432
ntpG	5.348
ndoS	5.328
SpyM3_1786	5.307
nrdG	5.284
salY	5.259
tal	5.240
SpyM3_1829	5.220
SpyM3_1792	5.206
SpyM3_1193	5.099
mscL	5.093
glpF.1	5.084
SpyM3_1828	5.032
hyl	5.024
SpyM3_1298	5.020
SpyM3_1752	4.987
lacF	4.950
ska	4.899
SpyM3_0999	4.820
citX	4.792
SpyM3_0187	4.676
SpyM3_0744	4.606
citF	4.581
SpyM3_1203	4.526
SpyM3_0743	4.480
SpyM3_1750	4.460
ugl	4.458
ntpI	4.437
hasA	4.423
ntpE	4.413
SpyM3_0366	4.395
malQ	4.392
citE	4.362
fdhC	4.329
scrA	4.328
adh1	4.273
SpyM3_0489	4.264
SpyM3_1493	4.258
SpyM3_1699	4.238
sdn	4.171
lacG	4.157
lctO	4.086
SpyM3_1751	4.000
glgP	3.975
glpO	3.967
SpyM3_1764	3.959
oadA	3.939
SpyM3_1743	3.914
arsC	3.882
SpyM3_1793	3.856
arcB	3.845
SpyM3_1197	3.808
SpyM3_1120	3.783
SpyM3_1105	3.778
ntpB	3.774
SpyM3_0139	3.774
SpyM3_1019	3.763
citD	3.710
gldA	3.707
mipB	3.698
pflD	3.698
SpyM3_1297	3.690
SpyM3_0894	3.685
SpyM3_0851	3.683
SpyM3_0677	3.670
ntpK	3.667
SpyM3_0769	3.651
slo	3.578
SpyM3_0742	3.545
ntpD	3.543
SpyM3_0114	3.500
lacC.2	3.474
agaW	3.473
SpyM3_0103	3.398
SpyM3_0789	3.397
nga	3.342

SpyM3_0748	3.285
SpyM3_0138	3.276
lacB.2	3.275
adh2	3.219
sclA_1	3.202
SpyM3_0129	3.201
SpyM3_0788	3.143
SpyM3_1714	3.105
glpK	3.098
nox.3	3.054
ahpC	3.030
agaV	3.026
scpA	3.017
SpyM3_1830	3.010
SpyM3_1254	3.008
hit	3.005
SpyM3_0323	2.996
SpyM3_0512	2.994
SpyM3_1583	2.937
SpyM3_1252	2.924
SpyM3_0038	2.924
trpG	2.910
SpyM3_1626	2.901
SpyM3_0102	2.895
SpyM3_0856	2.884
SpyM3_1339	2.876
SpyM3_0307	2.875
SpyM3_0857	2.874
SpyM3_0831	2.840
SpyM3_1259	2.824
SpyM3_0768	2.810
lplA	2.800
msmK	2.797
pfl	2.780
recT	2.780
SpyM3_0404	2.767
bglG	2.754
SpyM3_1250	2.724
SpyM3_0678	2.721
SpyM3_1445	2.716
msrA.1	2.710
SpyM3_1257	2.710
SpyM3_0408	2.707
SpyM3_0829	2.704
SpyM3_1268	2.693
SpyM3_0746	2.689
SpyM3_0099	2.684
aroE.1	2.665
SpyM3_0855	2.654
SpyM3_0101	2.630
punA	2.629
deaD.2	2.624
SpyM3_1499	2.623
SpyM3_0747	2.601
lacA.2	2.598
bglA.1	2.573
SpyM3_1446	2.560
SpyM3_1246	2.547
SpyM3_1249	2.540
SpyM3_1358	2.502
scrB	2.501
agaD	2.496
rpiA	2.485
SpyM3_1447	2.468
SpyM3_0870	2.456
lacD.2	2.455
SpyM3_0745	2.444
SpyM3_0535	2.444
SpyM3_1330	2.443
pblA	2.430
SpyM3_0741	2.429
SpyM3_0100	2.424
SpyM3_1240	2.417
SpyM3_0368	2.415
pflC	2.413
SpyM3_1505	2.413
SpyM3_0858	2.408
SpyM3_1444	2.388
SpyM3_1258	2.384
deoB	2.380
cbp	2.368
dpr	2.348
SpyM3_0107	2.332

SpyM3_1705	2.321
pabP	2.321
SpyM3_0257	2.288
SpyM3_1195	2.287
pepD	2.285
SpyM3_0534	2.282
folK	2.278
SpyM3_1452	2.277
SpyM3_1206	2.270
scrR	2.261
SpyM3_0367	2.258
SpyM3_0349	2.255
SpyM3_1335	2.254
SpyM3_1623	2.247
SpyM3_0383	2.246
SpyM3_0457	2.241
SpyM3_0384	2.241
SpyM3_1710	2.229
SpyM3_0142	2.227
SpyM3_0462	2.223
SpyM3_1200	2.201
SpyM3_0148	2.201
mutT	2.198
SpyM3_1473	2.198
atoE	2.190
SpyM3_0917	2.184
SpyM3_0900	2.183
SpyM3_1546	2.175
fmt	2.170
SpyM3_0520	2.163
SpyM3_1538	2.161
SpyM3_1270	2.156
salK	2.136
SpyM3_1207	2.135
copA	2.134
gloA	2.108
SpyM3_0442	2.106
SpyM3_1241	2.104
SpyM3_1497	2.102
folQ	2.095
SpyM3_1449	2.086
SpyM3_0351	2.082
SpyM3_1450	2.077
SpyM3_1248	2.075
SpyM3_1052	2.066
SpyM3_0490	2.066
SpyM3_0860	2.063
int315.6	2.058
SpyM3_1711	2.056
SpyM3_1100	2.054
recU	2.048
SpyM3_0382	2.046
int315.4	2.043
SpyM3_1454	2.041
SpyM3_1299	2.029
cycD	2.028
SpyM3_1283	2.026
SpyM3_0113	2.020
salR	2.020
argR	2.017
yesN	2.011
SpyM3_1056	2.011
polA.2	2.008
SpyM3_0108	2.007
SpyM3_0659	2.007
SpyM3_0615	2.006
purN	-2.000
rpsD	-2.000
dnaB	-2.001
relA	-2.003
citC	-2.004
SpyM3_0605	-2.017
SpyM3_1009	-2.019
SpyM3_0223	-2.019
mesJ	-2.021
SpyM3_0400	-2.022
gluS	-2.025
comYB	-2.028
prfB	-2.030
SpyM3_0553	-2.031
SpyM3_0603	-2.034
rplV	-2.038
SpyM3_0675	-2.038

snf	-2.040
mutY	-2.040
murC.1	-2.040
pdxK	-2.041
SpyM3_1365	-2.045
rplN	-2.052
pyk	-2.054
apt	-2.061
recR	-2.061
ftsA	-2.064
SpyM3_0398	-2.070
pbp2X	-2.071
era	-2.072
rpsC	-2.073
hsdM	-2.086
SpyM3_0632	-2.089
pyrH	-2.091
gcp	-2.092
pgi	-2.093
SpyM3_1221	-2.093
hsdR	-2.095
SpyM3_0568	-2.095
SpyM3_0072	-2.100
plr	-2.101
SpyM3_0838	-2.102
radA	-2.106
SpyM3_1275	-2.107
speK	-2.112
SpyM3_1500	-2.113
dyr	-2.113
SpyM3_0645	-2.117
SpyM3_0402	-2.119
SpyM3_0672	-2.119
SpyM3_1547	-2.122
SpyM3_0670	-2.123
ftsH	-2.124
SpyM3_1360	-2.128
SpyM3_0631	-2.128
rsuA	-2.140
SpyM3_0202	-2.148
fhuC.1	-2.150
rpmE	-2.150
SpyM3_1675	-2.156
SpyM3_0344	-2.164
divIB	-2.165
SpyM3_0397	-2.174
metB	-2.175
leuS	-2.179
SpyM3_0674	-2.184
clpX	-2.192
murN	-2.197
rpmH	-2.198
clpP.1	-2.200
SpyM3_1618	-2.204
hemN	-2.206
hlyX	-2.208
rplR	-2.211
SpyM3_1510	-2.212
SpyM3_0427	-2.214
ftsY	-2.215
SpyM3_1667	-2.216
amiC	-2.220
recF	-2.223
gidA	-2.229
mecA	-2.241
SpyM3_0224	-2.241
SpyM3_0231	-2.255
SpyM3_0811	-2.260
SpyM3_1607	-2.262
SpyM3_1810	-2.269
ddlA	-2.278
pyrP	-2.279
SpyM3_0778	-2.282
SpyM3_0572	-2.289
nifS	-2.290
cysE	-2.297
SpyM3_0477	-2.303
SpyM3_0338	-2.304
SpyM3_0389	-2.305
rpsF	-2.317
SpyM3_1094	-2.321
SpyM3_0542	-2.321
hsdS	-2.325

pgk	-2.328
SpyM3_0641	-2.329
SpyM3_0356	-2.339
pyrE	-2.351
lmb	-2.352
acpA	-2.355
rgpG	-2.355
rplD	-2.356
murM.1	-2.362
SpyM3_1479	-2.369
SpyM3_1186	-2.382
SpyM3_1845	-2.384
truB	-2.390
SpyM3_1187	-2.390
rnpA	-2.394
SpyM3_1671	-2.396
SpyM3_1799	-2.397
rpsM	-2.401
SpyM3_1849	-2.410
SpyM3_1017	-2.410
adk	-2.412
SpyM3_1715	-2.419
SpyM3_1398	-2.420
SpyM3_0848	-2.424
SpyM3_1393	-2.424
rplX	-2.430
SpyM3_0946	-2.433
SpyM3_0259	-2.434
SpyM3_0013	-2.435
kdtB	-2.450
SpyM3_1591	-2.454
SpyM3_0417	-2.456
SpyM3_0295	-2.461
SpyM3_1177	-2.479
tsf	-2.481
SpyM3_1361	-2.486
prfC	-2.490
pepXP	-2.490
comFC	-2.493
purK	-2.494
SpyM3_1627	-2.502
SpyM3_0499	-2.507
rpsE	-2.508
exoA	-2.514
SpyM3_0225	-2.516
rluD	-2.517
SpyM3_0243	-2.522
SpyM3_1081	-2.523
folD	-2.524
rpmI	-2.536
rpsK	-2.539
rplB	-2.547
SpyM3_1170	-2.555
rplT	-2.555
SpyM3_0850	-2.558
prgA	-2.565
pyrD	-2.566
SpyM3_0087	-2.566
ung	-2.570
nrdE.1	-2.577
SpyM3_0340	-2.583
gatC	-2.592
SpyM3_0271	-2.604
phr	-2.606
clpE	-2.612
SpyM3_0425	-2.616
inlA	-2.642
opuABC	-2.645
thiI	-2.647
SpyM3_1302	-2.650
SpyM3_1278	-2.666
SpyM3_1662	-2.673
rpmG	-2.674
SpyM3_0756	-2.680
SpyM3_0422	-2.688
rplS	-2.708
SpyM3_1069	-2.712
SpyM3_1787	-2.713
ssb.2	-2.728
SpyM3_0227	-2.730
SpyM3_0288	-2.730
SpyM3_0557	-2.734
ropA	-2.739

SpyM3_0823	-2.739
SpyM3_0226	-2.742
SpyM3_1021	-2.748
mtsB	-2.759
SpyM3_0168	-2.764
ctsR	-2.765
ftsW	-2.766
ropB	-2.768
SpyM3_1834	-2.774
SpyM3_1277	-2.779
SpyM3_0423	-2.783
SpyM3_0541	-2.786
aapA	-2.793
SpyM3_0660	-2.798
mraW	-2.802
parE	-2.807
guaA	-2.808
SpyM3_0176	-2.822
SpyM3_0890	-2.829
SpyM3_1579	-2.836
SpyM3_0565	-2.841
SpyM3_1076	-2.842
SpyM3_1020	-2.847
parC	-2.854
SpyM3_0497	-2.855
SpyM3_0785	-2.858
rplJ	-2.859
pyrC	-2.860
SpyM3_1397	-2.863
SpyM3_1185	-2.874
rpsB	-2.879
SpyM3_0591	-2.880
SpyM3_1075	-2.883
SpyM3_0230	-2.889
SpyM3_1673	-2.902
SpyM3_0203	-2.904
SpyM3_0421	-2.908
SpyM3_0229	-2.918
SpyM3_0254	-2.931
SpyM3_0889	-2.938
SpyM3_0784	-2.942
SpyM3_1368	-2.953
pcp	-2.958
nadE	-2.959
SpyM3_1396	-2.959
SpyM3_0177	-2.964
prfA	-2.968
pyrF	-2.968
ftsL	-2.975
trsA	-2.986
SpyM3_1846	-2.993
emm3	-3.007
fusA	-3.021
smc	-3.034
lsp	-3.043
gyrA	-3.081
SpyM3_0888	-3.094
cysS	-3.101
SpyM3_0843	-3.105
SpyM3_1514	-3.106
rplC	-3.106
SpyM3_1068	-3.111
covS	-3.125
nth	-3.131
SpyM3_1183	-3.132
SpyM3_1388	-3.144
purE	-3.147
secY	-3.154
SpyM3_0644	-3.170
ftsX	-3.177
rpsI	-3.178
hpt	-3.184
SpyM3_1122	-3.185
SpyM3_0085	-3.188
tyrS	-3.194
mvaS.1	-3.203
grab	-3.205
SpyM3_0493	-3.209
purD	-3.211
eno	-3.214
trmD	-3.221
SpyM3_1067	-3.223
gapN	-3.231

topA	-3.244
SpyM3_1070	-3.251
SpyM3_1850	-3.301
SpyM3_0582	-3.304
mtsA	-3.309
tufA	-3.326
SpyM3_1578	-3.332
SpyM3_0228	-3.335
SpyM3_0755	-3.335
greA	-3.343
SpyM3_t59	-3.346
pyrR	-3.358
SpyM3_1631	-3.366
ylxM	-3.369
queA	-3.371
SpyM3_0555	-3.376
SpyM3_0783	-3.389
SpyM3_1860	-3.398
rplA	-3.402
SpyM3_1077	-3.408
rpmB	-3.438
SpyM3_0437	-3.444
SpyM3_0627	-3.462
cadX	-3.471
nagB	-3.476
rpsJ	-3.483
infA	-3.484
murM.2	-3.486
SpyM3_0847	-3.490
trmU	-3.492
tpi	-3.509
pfk	-3.527
SpyM3_1839	-3.540
spoJ	-3.551
SpyM3_0606	-3.556
SpyM3_1848	-3.561
SpyM3_1178	-3.575
SpyM3_0630	-3.584
kup	-3.607
glpF.2	-3.616
SpyM3_1844	-3.618
infC	-3.626
gidB	-3.631
SpyM3_0812	-3.633
SpyM3_0840	-3.693
SpyM3_0844	-3.757
SpyM3_0357	-3.759
SpyM3_0272	-3.771
SpyM3_0669	-3.771
thdF	-3.833
SpyM3_1507	-3.853
rpmF	-3.883
SpyM3_1015	-3.889
SpyM3_0358	-3.955
mvaS.2	-3.957
SpyM3_0328	-3.967
oppA	-3.985
SpyM3_0902	-4.013
SpyM3_1665	-4.018
upp	-4.031
guaB	-4.051
SpyM3_0273	-4.056
SpyM3_1509	-4.095
rplM	-4.125
rpsN.2	-4.148
rpsG	-4.150
dnaQ	-4.174
SpyM3_0643	-4.187
cadD	-4.201
SpyM3_1602	-4.208
rpoA	-4.224
rplK	-4.238
SpyM3_1160	-4.254
SpyM3_0003	-4.262
rimM	-4.268
trx.1	-4.301
opuAA	-4.324
nrdH	-4.369
deaD.1	-4.393
pgsA	-4.399
dut	-4.409
SpyM3_0299	-4.428
SpyM3_1508	-4.472

SpyM3_0652	-4.477
ffh	-4.508
SpyM3_1184	-4.556
tdk	-4.559
gmk	-4.589
glnQ	-4.595
SpyM3_0330	-4.601
rpsL	-4.641
SpyM3_0656	-4.671
pepC	-4.733
SpyM3_0424	-4.740
SpyM3_1374	-4.820
msrA.2	-4.855
glnP	-5.291
SpyM3_1599	-5.317
SpyM3_0104	-5.325
SpyM3_0906	-5.354
SpyM3_0494	-5.528
SpyM3_1060	-5.619
SpyM3_0908	-5.663
SpyM3_1061	-5.689
SpyM3_0655	-5.718
SpyM3_0796	-5.735
SpyM3_0907	-5.739
SpyM3_0090	-6.033
SpyM3_0797	-6.085
degP	-6.101
SpyM3_0092	-6.132
SpyM3_0258	-6.133
bcaT	-6.137
SpyM3_1040	-6.139
SpyM3_0793	-6.355
SpyM3_0439	-6.430
purC	-6.628
SpyM3_0628	-6.682
SpyM3_0653	-6.940
SpyM3_1161	-7.386
SpyM3_0014	-7.586
udk	-7.870
SpyM3_1041	-8.806
asnA	-9.462
SpyM3_0795	-9.675
SpyM3_0909	-10.308
SpyM3_0237	-11.710
SpyM3_1843	-11.917
SpyM3_0589	-12.355
braB	-12.654
fms	-12.778
SpyM3_0654	-14.000
xpt	-14.627

Table S4

Genes differentially regulated between our parental M3 isolate and its derivative M3rocA^{COMP}. Color-coding is consistent with that shown in figure 8C.
